# π-Stacking Stopper-Macrocycle Stabilized Dynamically Interlocked [2]Rotaxanes

**DOI:** 10.3390/molecules26154704

**Published:** 2021-08-03

**Authors:** Sing-Ming Chan, Fung-Kit Tang, Ching-Yau Lam, Chak-Shing Kwan, Sam C. K. Hau, Ken Cham-Fai Leung

**Affiliations:** 1 State Key Laboratory of Environmental and Biological Analysis, Department of Chemistry, The Hong Kong Baptist University, Kowloon Tong, Kowloon, Hong Kong, China; 17482100@life.hkbu.edu.hk (S.-M.C.); 18482503@life.hkbu.edu.hk (F.-K.T.); 16213769@life.hkbu.edu.hk (C.-Y.L.); 14485761@life.hkbu.edu.hk (C.-S.K.); 2 Department of Chemistry, The Chinese University of Hong Kong, Shatin, NT, Hong Kong, China

**Keywords:** dynamic combinatorial library, imine macrocycle, intercomponent interaction, π-stacking, rotaxane synthesis

## Abstract

The synthesis of mechanically interlocked molecules is valuable due to their unique topologies. With π-stacking intercomponent interaction, e.g., phenanthroline and anthracene, novel [2]rotaxanes have been synthesized by dynamic imine clipping reaction. Their X-ray crystal structures indicate the π-stackings between the anthracene moiety (stopper) on the thread and the (hetero)aromatic rings at the macrocycle of the rotaxanes. Moreover, the length of glycol chains affects the extra π-stacking intercomponent interactions between the phenyl groups and the dimethoxy phenyl groups on the thread. Dynamic combinatorial library has shown at best 84% distribution of anthracene-threaded phenanthroline-based rotaxane, coinciding with the crystallography in that the additional π-stacking intercomponent interactions could increase the thermodynamic stability and selectivity of the rotaxanes.

## 1. Introduction

The synthesis of mechanically interlocked molecules, such as [*n*]rotaxanes and [*n*]catenanes [1,2,3,4], has drawn interest due to their unique topologies. They have been applied in the designs of molecular machinery [5,6,7,8] and molecular sensing [9,10,11,12,13,14]. For the [*n*]rotaxanes, templated-direct syntheses using secondary dialkylammonium (R_2_NH_2_^+^) ions are involved in threading followed by stoppering [15], slippage [16,17,18,19] and clipping approaches [20,21] Various macrocycles, such as dibenzo[24]crown-8 (DB24C8) and benzo[21]crown-7 (B21C7), can be threaded through by the dialkylammonium (R_2_NH_2_^+^) ions with sufficiently high binding constant [22,23,24].

In 2001, Stoddart and co-workers developed the dynamic clipping on R_2_NH_2_^+^ ions for [2]rotaxane synthesis [25]. As being part of the dynamic covalent chemistry (DCC) [21,26,27], the dynamic clipping approach allows self-error checking and self-sorting to favor the most thermodynamically stable rotaxane as the major product based on equilibrium reactions. Besides [2]rotaxanes, efficient methods for constructing dendritic [*n*]rotaxanes [28,29,30,31,32] homocircuit [*n*]rotaxanes [33,34,35,36,37,38] and heterocircuit [*n*]rotaxanes [39,40,41,42,43,44,45] have recently been investigated.

Meanwhile, the synthetic complexity could raise if the number of recognition sites increases or the thread became unsymmetrical. Indeed, the co-conformational [2]rotaxane isomers should have good stability to avoid isomerization by molecular shuttling. Therefore, developing a selective synthesis of dynamic [2]rotaxane with only one of R_2_NH_2_^+^ ions recognition site occupied by macrocycle is highly critical.

Herein, we report new phenanthroline-based rotaxanes by employing 1,10-phenanthroline-2,9-dicarbaldehyde (dialdehyde **1**) to form dynamic imine macrocycle. In the presence of the anthracene moiety, additional π-stacking between the phenanthroline unit and anthracene unit is observed in the solid-state structures of [2]rotaxanes, indicating further stabilization of the interlocked structure. The π-stacking at the stopper offers a higher specificity on rotaxane synthesis, making anthracene threaded phenanthroline-based rotaxanes as the major products in dynamic combinatorial library competing with other threads.

## 2. Results and Discussion

The 2,6-pyridine dicarboxaldehyde (dialdehyde **2**) and tetraethylene glycol bis(2-aminophenyl)ether (diamine **3**) were used for the synthesis of rotaxanes [14,25,36,46] by the clipping approach. For instance, **10**-H·PF_6_ and its stability in the presence of water and acid were reported [47]. As the stopper of rotaxanes, the anthracene unit was adopted due to its fluorescence property and bulkiness. Although the crystal structures of [3]rotaxane [36,42] nickel(II)–salen [2]rotaxane [48,49] and triimine [2]rotaxanes [50,51] had been previously reported, single crystals of pure organic diimine [2]rotaxane were not discovered yet.

**Scheme 1:**
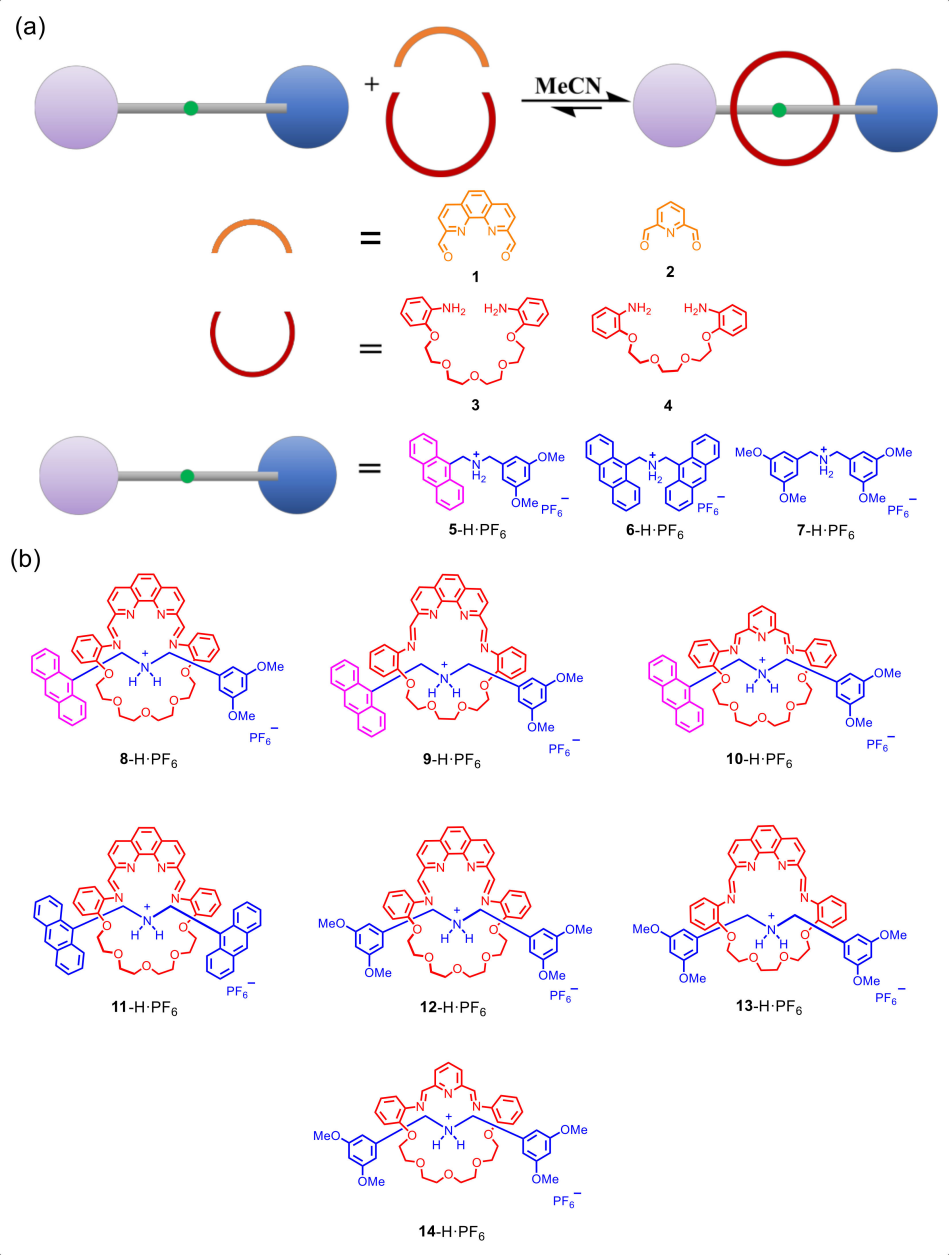
(**a**) Synthesis of dynamic [2]rotaxanes by clipping of dialdehydes (**1** or **2**), diamines (**3** or **4**), and threads (**5**-H·PF_6_, **6**-H·PF_6_ or **7**-H·PF_6_); (**b**) [2]rotaxanes obtained from synthesis in (**a**).

**Figure 1:**
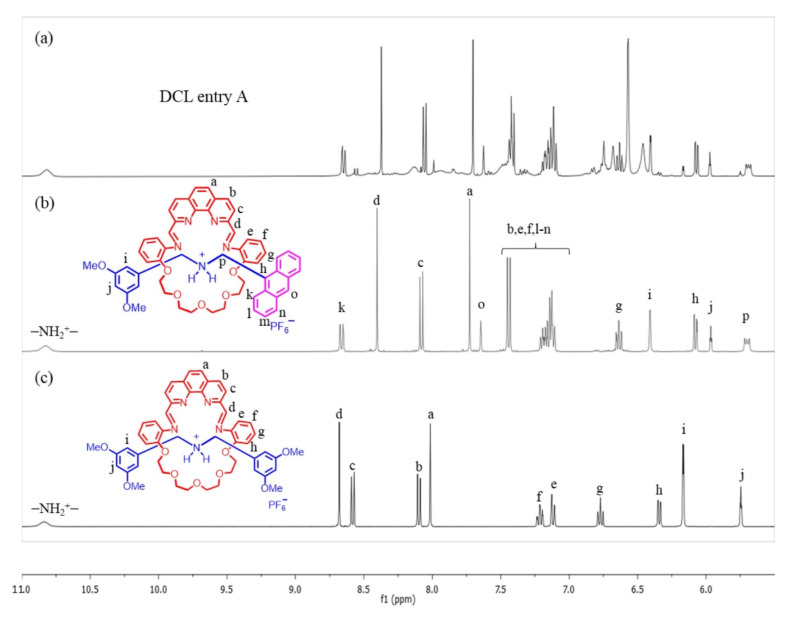
Partial, stacked ^1^H NMR spectra (CD_3_CN, 400 MHz, 298 K) of (**a**) dynamic combinatorial library (DCL) entry A; (**b**) **8**-H·PF_6_; and (**c**) **12**-H·PF_6_.

To verify the intercomponent stabilization [52,53,54,55,56,57], rotaxanes with stronger π-stacking interaction were synthesized. Dialdehyde **1** was selected due to its conjugated aromatic structure. By mixing equimolar amounts of thread **5**-H·PF_6_, dialdehyde **1** and diamine **3** in MeCN at room temperature (Scheme 1), the crude ^1^H NMR spectrum (Figure 1b) of the reaction mixtures reveals total consumption of the starting materials to the respective [2]rotaxane, **8**-H·PF_6_. The disappearance of the aldehyde proton –C***H***O resonance at ca. 10.2 ppm supports the complete consumption of the dialdehyde compound in the reaction. Meanwhile, the characteristic signal for the benzylic methylene group –C***H*_2_** protons adjacent to the R_2_NH_2_^+^ center was shifted and split from *δ* = 5.2 and 4.4 ppm in thread **5**-H·PF_6_ to *δ* = 5.75–5.64 and 4.72–4.60 ppm in rotaxane **8**-H·PF_6_, indicating its encirclement by the crown ether rings (Table S1). Hence, the macrocycle formed in the reaction contains two more carbon atoms and one more nitrogen atom compared to that of dialdehyde **2**, making **8**-H·PF_6_ a [27]crown-9 rotaxane. Moreover, by changing the diamine from **3** to **4**, [24]crown-8 rotaxane **9**-H·PF_6_ was obtained in high yield. For dialdehyde **2**, only [24]crown-8 rotaxane **10**-H·PF_6_ and **14**-H·PF_6_ were obtained possibly due to the weaker π–π stacking stabilization.

**Figure 2:**
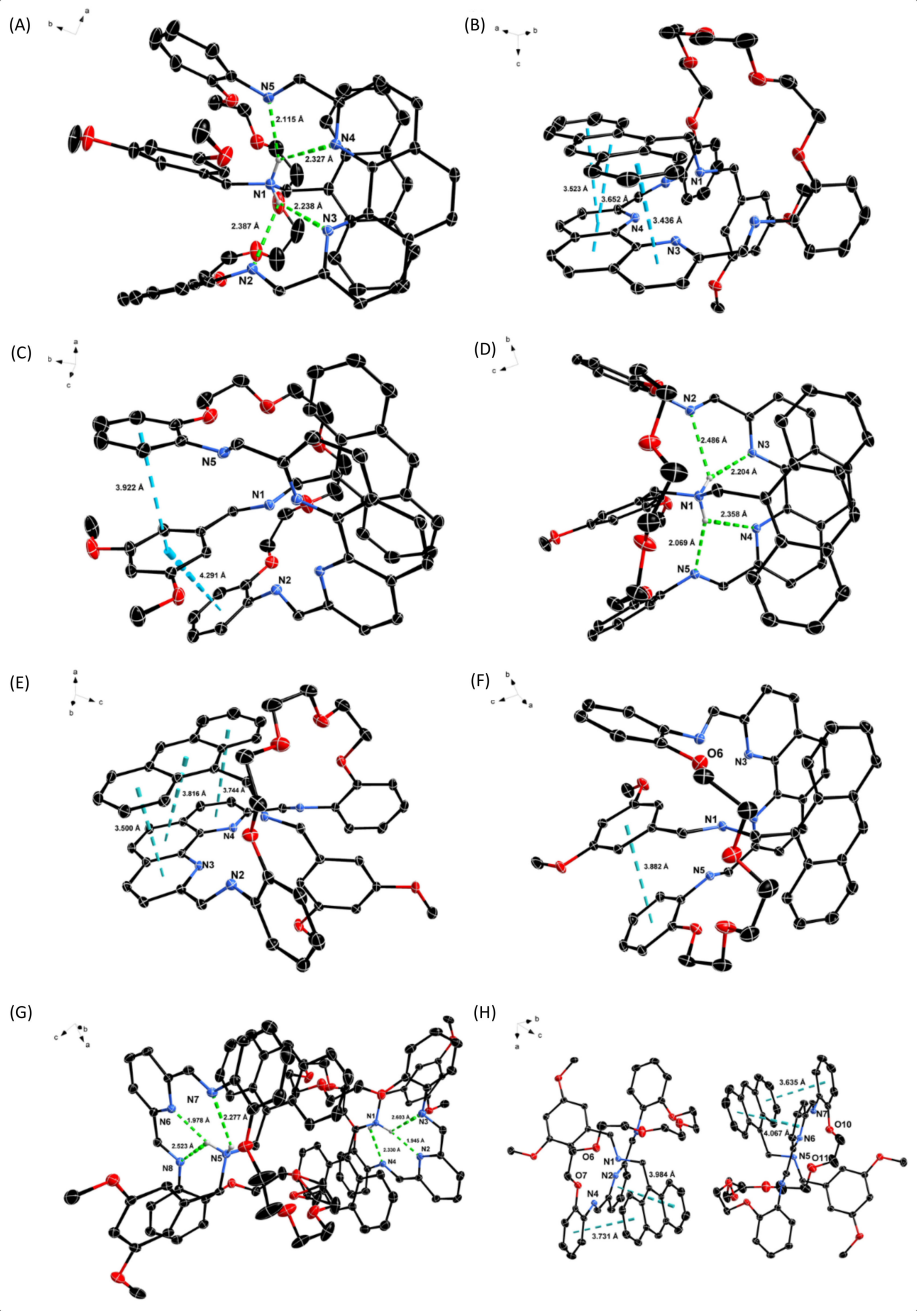
Perspective view of (**A**) solid-state structures of dynamic rotaxane **8**-H·PF_6_ (CCDC code: 1993136); (**B**) the offset face-to-face π–π stacking intercomponent interaction between the anthracene and phenanthroline in **8**-H·PF_6_; (**C**) the offset face-to-face π–π stacking intercomponent interaction between the phenyl moieties in **8**-H·PF_6_; (**D**) solid-state structures of dynamic rotaxane **9**-H·PF_6_ (CCDC code: 1993139); (**E**) the offset face-to-face π–π stacking intercomponent interaction between the anthracene and phenanthroline in **9**-H·PF_6_; (**F**) the offset face-to-face π–π stacking intercomponent interaction between the phenyl moieties in **9**-H·PF_6_; (**G**) solid-state structures of dynamic rotaxane **10**-H·PF_6_ (CCDC code: 1993140); (**H**) the offset face-to-face π–π stacking intercomponent interaction between the anthracene and pyridine in **10**-H·PF_6_. All hydrogen atoms and PF_6_^–^ anions are omitted for clarity.

To study the π-stacking intercomponent interaction in the phenanthroline-based rotaxanes, the rotaxanes were crystallized for X-ray crystallography analysis. Single crystals of **8**-H·PF_6_ and **9**-H·PF_6_ were obtained by slow evaporation from CH_2_Cl_2_-hexane solution and MeCN-*^i^*Pr_2_O solution, respectively. From the solid-state structures (Figure 2A–F, Table S2), [N^+^–H⋅⋅⋅N] interaction was observed between the dialkylammonium ion, diimine and the phenanthroline moiety in both rotaxanes. Interestingly, the ethylene glycol unit (−*CH*_2_*CH*_2_*O−*) did not interact with the dialkylammonium ion as [N^+^–H⋅⋅⋅O] in **8**-H·PF_6_, **9**-H·PF_6_ and **10**-H·PF_6_, which is an essential interaction in DB24C8- and B21C7-based rotaxane was not observed. Moreover, π-stacking intercomponent interactions were observed between the anthracene and phenanthroline unit with center-to-center distance ranges from 3.437 to 3.652 Å in **8**-H·PF_6_ and from 3.500 to 3.816 Å in **9**-H·PF_6_, respectively. The two phenyl moieties participate in the π-stacking intercomponent interaction with another stopper, the 3,5-dimethoxylphenyl unit. The center-to-center distance between the three phenyl rings for **8**-H·PF_6_ is 3.922 Å and 4.291 Å while the center-to-center distance between the two phenyl rings for **9**-H·PF_6_ is 3.882 Å. The solid-state structures suggest that the π-stacking between the anthracene and the phenanthroline possesses a stronger interaction which can further stabilize the [2]rotaxanes. Moreover, the phenanthroline unit is taking a more important role than the ethylene glycol units on formation of the [2]rotaxanes.

By slow evaporation of the MeCN solution of **10**-H·PF_6_, single crystal was obtained and analyzed by X-ray crystallography. In addition to the [N^+^–H⋅⋅⋅N] interaction, the solid-state structure of **10**-H·PF_6_ (Figure 2G,H) shows aromatic π–π stacking interactions between the anthracene and phenyl unit with center to center π–π stacking interaction distance: 3.636 Å. With the π-stacking intercomponent interactions between the stopper and the macrocycle, the anthracene thread can be stabilized the rotaxane formation and allows the isolation of rotaxanes.

In order to test the compatibility of the formation of dynamic rotaxanes, other stoppers for clipping were used. In particular, threads **6**-H·PF_6_ and **7**-H·PF_6_ were used to investigate the effect of the π-stacking interactions, while clipping with thread **7**-H·PF_6_ gave symmetrical rotaxanes **12**-H·PF_6_ and **13**-H·PF_6_ in high yields. ^1^H NMR signals of the phenanthroline unit in **12**-H·PF_6_ and **13**-H·PF_6_ have a significant difference in comparison to the signals in **8**-H·PF_6_ and **9**-H·PF_6_ (Figure 1 and Figure S17, Table S1). For **8**-H·PF_6_, H_a_, H_b_, H_c_ and H_d_ were upfield-shifted from *δ* = 8.01 to 7.73 ppm, *δ* = 8.10 to 7.44 ppm, *δ* = 8.58 to 8.08 ppm and *δ* = 8.68 to 8.40 ppm, respectively, compared to **12**-H·PF_6_. The upfield movement of phenanthroline aromatic proton signals of **8**-H·PF_6_ and **9**-H·PF_6_ can be due to the electron sharing from the electron-rich anthracene moiety. Moreover, phenanthroline aromatic proton was located in anisotropic-induced magnetic field of the anthracene moiety, so it is upfield shifted because of shielding effect. Both support the existence of the π-stacking interactions. For thread **6**-H·PF_6_, only rotaxane **11**-H·PF_6_ was obtained in low yield; changing the components to dialdehyde **2** or diamine **4** did not show a reasonable structure of rotaxane. This might be due to the strong intramolecular π-stacking between the two anthracene stoppers, so thread **6**-H·PF_6_ was folded and not reactive to form dynamic rotaxane. As a result, dialdehyde **1** allows more comprehensive combination to form dynamic rotaxane compared to dialdehyde **2**, showing the potential of using phenanthroline for dynamic rotaxane synthesis.

**Scheme 2:**
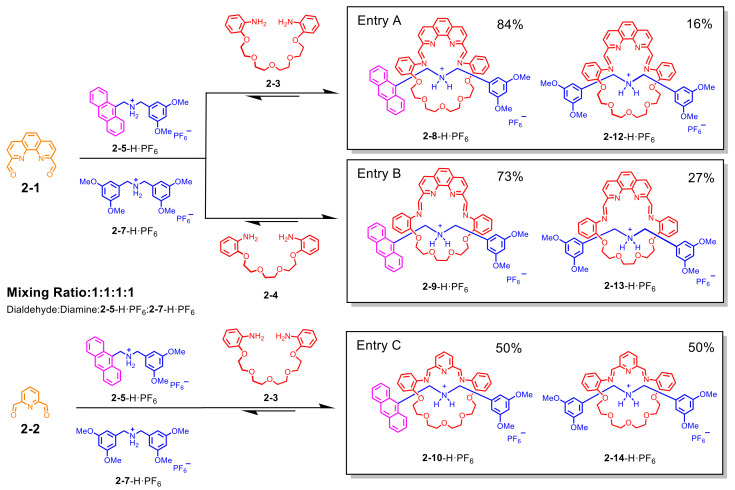
Outcome of DCL entries A-C, by clipping of the dialdehydes **1** or **2** and diamine **3** or **4** with threads **5**-H·PF_6_ and **7**-H·PF_6_ to form the [2]rotaxanes.

After the successful synthesis of five new phenanthroline-based rotaxanes, dynamic combinatorial library (DCL) experiments were conducted to study the effect of π-stacking stabilization (Scheme 2). Threads **5**-H·PF_6_ and **7**-H·PF_6_ in 1:1 ratio were mixed with different dialdehydes and diamines in the reaction. As expected, there should be a competition on forming either anthracene-based or symmetrical rotaxane. The dominant product in each experimental entry should be more thermodynamically stable. Moreover, the temperature was set to 50 °C to maximize the thermodynamic selectivity of rotaxanes. After reacting for 18 h, ratios of rotaxanes were determined by integrating the proton signals in the ^1^H NMR spectra (Figure 1, Figures S17 and S18). As a result, ^1^H NMR spectrum of dynamic combinatorial library experiment indicates that the thread **5**-H·PF_6_ was more favorable than **7**-H·PF_6_ to undergo rotaxane clipping with dialdehyde **1**. **8**-H·PF_6_ and **9**-H·PF_6_ were preferentially formed as the dominant products, which have an 84:16 and 73:27 NMR yield ratios compared to the **12**-H·PF_6_ and **13**-H·PF_6_, respectively (Table 1). Moreover, the better selectivity in Entry A compared to Entry B may be due to the enhanced effect of the π-stacking interaction between the dimethyoxyphenyl moiety on stopper and the phenyl moiety on the macrocycle. While there was no preference for the synthesis of **10**-H·PF_6_ and **14**-H·PF_6_, 50:50 NMR yield ratio was obtained in entry C using the pyridine dialdehyde **2**. The reason is that the weak π-stacking interaction did not affect the stability of rotaxanes significantly, which further gives evidence of the phenanthroline unit in providing additional π-stacking interaction between the thread’s stopper and the macrocycle.

## 3. Materials and Methods

Instrumentation and General experimental. Unless otherwise stated, all reagents and anhydrous solvents were purchased from commercial sources and used without further purification. Acetonitrile was distilled over calcium hydride. Dried acetonitrile was stored over 3 Å molecular sieves. Flash column chromatography was performed using silica gel or aluminum oxide. Analytical TLC was performed on pre-coated silica gel plates (0.25 mm thick, 60F254, Merck, Darmstadt, Germany) and pre-coated ALUGRAM^®^ (0.20 mm thick, F254, MN, Dueren, Germany). Spots were observed under UV light. ^1^H and ^13^C NMR spectra were recorded on a Bruker Advance-III spectrometer (at 400 and 101 MHz, respectively). Chemical shifts are reported in parts per million from low to high field and referenced to residual solvent (CDCl_3_: ^1^H, 7.26 ppm; ^13^C, 77.16 ppm. CD_3_CN: ^1^H, 1.94 ppm; ^13^C, 1.32 ppm). NMR data were processed using MestReNova software (Mestrelab). Coupling constants are reported in Hertz. Standard abbreviations indicating multiplicity were used as follows: m = multiplet, quint = quintet, q = quartet, t = triplet, d = doublet, s = singlet, br = broad. High-resolution mass spectra were recorded on a Bruker Autoflex mass spectrometer (MALDI-TOF) and a Thermo Fisher Scientific UPLC-Q exactive focus hybrid quadrupole-orbitrap mass spectrometer in positive ion mode (ESI-MS). Selected crystals were used for intensity data collection on a Bruker AXS Kappa Apex II Duo diffractometer at 173K using frames of oscillation range 0.3°, with 2° < θ < 28°.

General procedure for synthesis of [2]rotaxanes. A solution of thread (0.1 mmol) in MeCN (15 mL) was added diamine (0.1 mmol) and dialdehyde (0.1 mmol). The yellow mixture was stirred for overnight at room temperature. The solvents were evaporated under reduced pressure. Product is isolated by recrystallization (MeCN/Et_2_O) or column chromatography (neutral alumina, CH_2_Cl_2_/*n*-hexane).

General procedure for dynamic combinatorial library entries A−C. A solution of thread **5**-H·PF_6_ (0.1 mmol) and thread **7**-H·PF_6_ (0.1 mmol) in MeCN (15 mL) was added a diamine (0.1 mmol), dialdehyde (0.1 mmol) and 0.1 g of 3 Å molecular sieve. The yellow mixture was stirred for 18 h at 50 °C. The solvents were evaporated under reduced pressure. The ratio of rotaxanes in mixture was measured by ^1^H NMR spectroscopy.

## 4. Conclusions

In conclusion, we had successfully synthesized new [2]rotaxanes by imine clipping reaction to form the macrocycle. π-Stacking intercomponent interactions between the macrocycle and the stopper of [2]rotaxanes **8**-H·PF_6_, **9**-H·PF_6_ and **10**-H·PF_6_ were confirmed from their X-ray structural analysis. Intercomponent interactions were deduced and also corroborated in solution, besides solid state. Dynamic combinatorial library experiments of rotaxane formation resulted in phenanthroline-based rotaxanes with anthracene unit in threads as the major products, demonstrating the importance of π-stacking intercomponent interaction between the stopper and the macrocycle on the selective synthesis of the dynamic rotaxanes or related imine-based organic-inorganic hybrid materials [58].

## Figures and Tables

**Table 1:** Dynamic combinatorial libraries (DCL) of rotaxane synthesis ^a^.

DCL Entry	Thread	Diamine	Dialdehyde	Products	Yields ^b^ (Ratio)
A	**5**-H·PF_6_ + **7**-H·PF_6_	**3**	**1**	**8**-H·PF_6_ + **12**-H·PF_6_	84:16
B	**5**-H·PF_6_ + **7**-H·PF_6_	**4**	**1**	**9**-H·PF_6_ + **13**-H·PF_6_	73:27
C	**5**-H·PF_6_ + **7**-H·PF_6_	**3**	**2**	**10**-H·PF_6_ + **14**-H·PF_6_	50:50

^a^ Reagents and conditions: 0.1 mmol of each thread, diamine, dialdehyde, 15 mL of MeCN, 0.1 g of 3 Å molecular sieve, 50 °C. ^b^ Ratios determined by ^1^H NMR spectroscopic analysis of the reaction mixture after 18 h.

## Data Availability

The data presented in this study are available on request from the corresponding authors.

## Supplementary Materials

The following are available online: Experimental data of new compounds and X-ray crystallography data.
