# Supplementary material for: π-Stacking Stopper-Macrocycle Stabilized Dynamically Interlocked [2]Rotaxanes
Source: Molecules. 2021 Aug 3;26(15):4704. doi: 10.3390/molecules26154704 (PMC8347712; doi:10.3390/molecules26154704)
Supplement: Supplementary file 1 [file molecules-26-04704-s001.zip › ESI.pdf]

Electronic Supplementary Information

π–Stacking Stopper-Macrocycle Stabilized Dynamically Interlocked [2]Rotaxanes

Sing-Ming Chan,[a] Fung-Kit Tang,[a] Ching-Yau Lam,[a] Chak-Shing Kwan,[a] Sam C. K. Hau*[b] and Ken Cham-Fai Leung*[a]

[a] Department of Chemistry, The Hong Kong Baptist University, Kowloon Tong, Kowloon, Hong Kong SAR, P. R. China
E-mail: cfleung@hkbu.edu.hk
[b] Department of Chemistry, The Chinese University of Hong Kong, Shatin, New Territories, Hong Kong SAR, P. R. China
E-mail: sckhau@cuhk.edu.hk

Synthesis

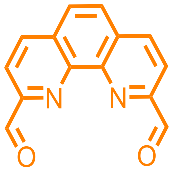

The synthesis of dialdehyde **1** was reported in the literature [1].

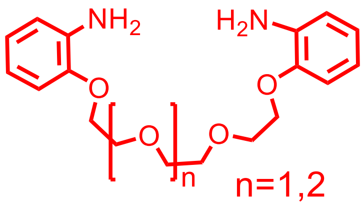

The synthesis of diamines **3** and **4** was reported in the literature [2].

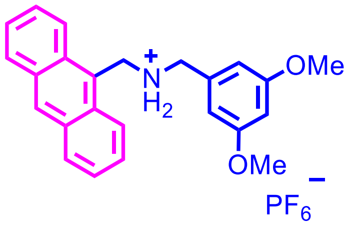

The synthesis of **5**-H·PF_6 was reported in the literature [3].

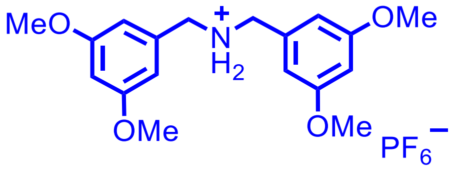

The synthesis of **7**-H·PF_6 was reported in the literature [3].

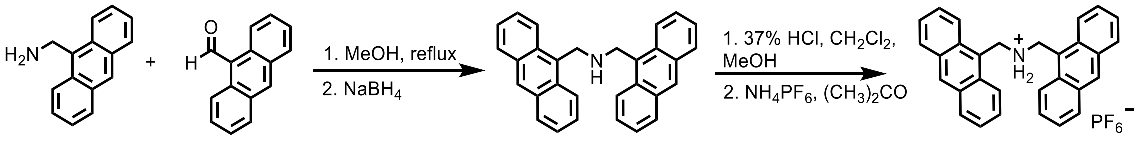

Scheme S1. Synthesis of the thread **6**-H·PF_6.

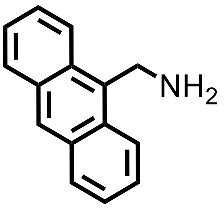

The synthesis of 9-aminomethylanthracene was reported in the literature [4].

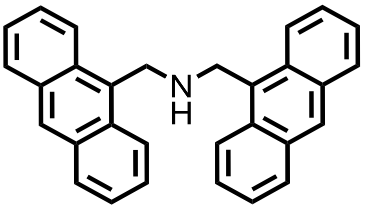

Synthesis of compound **6**:

A solution of 9-aminomethylanthracene (0.20 g, 0.98 mmol) in THF/MeOH (1:2, v/v, 30 mL) was added 9-anthracenecarboxaldehyde (0.20 g, 0.98 mmol) in THF/MeOH (1:2, v/v, 30 mL). The mixture was refluxed for overnight. Then the mixture was cooled to 0 °C and $NaBH_4$ (0.24 g, 6.34 mmol) was added. The mixture was refluxed for overnight. After that, solvent was evaporated under reduced pressure. The mixture was dissolved in EtOAc (100 mL) and washed with water (20 mL). The organic layer was dried with $MgSO_4$ and concentrated by evaporation. The crude product was purified by silica gel column chromatography with CH_2Cl_2 to afford **6** as a pale-yellow solid (0.22g, 56%).

^{1}H NMR (400 MHz, $CDCl_3$) δ 8.40 (s, 2H), 8.28 – 8.19 (m, 4H), 8.03 – 7.95 (m, 4H), 7.48 – 7.36 (m, 8H), 4.94 (s, 4H). NH signal is missing.

^{13}C NMR (101 MHz, $CDCl_3$) δ 131.62, 131.45, 130.55, 129.24, 127.39, 126.16, 125.02, 124.16, 45.71.

HRMS (ESI): cald. for $C_{30}H_{23}N$ $[M+H]^+$ *m/z* 398.1903, found 398.1890

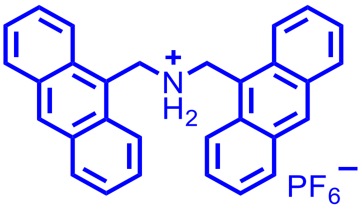

Synthesis of thread **6**-H·PF_6:

A solution of **6** (0.22 g, 0.55 mmol) in CH_2Cl_2/MeOH (10 mL) was added conc. HCl (2 mL). The mixture was stirred for 2 hours. Then the solvent was evaporated under reduced pressure. The mixture was dissolved in acetone (50 mL) and saturated NH_4PF_6 aqueous solution was added. The mixture was stirred for 2 hours. Then the organic solvent was evaporated under reduced pressure. The precipitate was filtered and washed with water to afford **6**-H·PF_6 as a pale-yellow solid (0.22g, 73%).

^{1}H NMR (400 MHz, CD_3CN) δ 8.62 (s, 2H), 8.23 – 8.17 (m, 4H), 8.12 – 8.04 (m, 4H), 7.58 – 7.47 (m, 8H), 5.25 (s, 4H). NH signal is missing.

^{13}C NMR (101 MHz, CD_3CN) δ 132.36, 131.67, 130.37, 130.31, 128.00, 126.43, 124.52, 45.53.

HRMS (ESI): cald. for $C_{30}H_{24}F_6NP$ $[M–PF_6]^+$ *m/z* 398.1903, found 398.1890

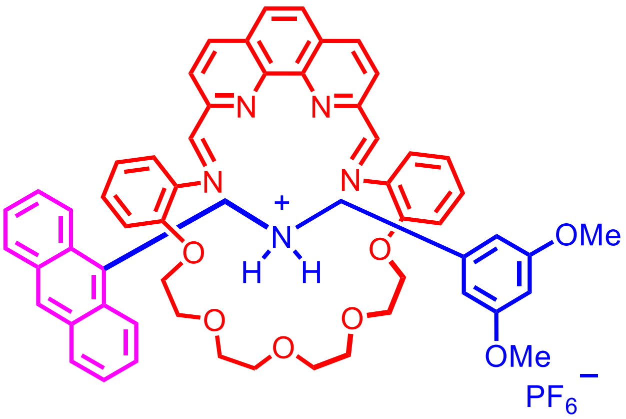

Synthesis of **8**-H·PF_6:

A solution of thread **5**-H·PF_6 (50.3 mg, 0.1 mmol) in MeCN (15 mL) was added diamine **3** (37.6 mg, 0.1 mmol) and dialdehyde **1** (23.6 mg, 0.1 mmol). The yellow mixture was stirred for overnight at room temperature. The solvents were evaporated under reduced pressure. The mixture was purified by neutral aluminum oxide column chromatography with (1/3, v/v) CH_2Cl_2/*n*-hexane, followed by CH_2Cl_2 to afford **8**-H·PF_6 as a pale-yellow powder (94.0 mg, 87%).

^{1}H NMR spectrum (400 MHz, CD_3CN) δ 10.82 (br, 2H), 8.66 (d, *J* = 8.7 Hz, 2H), 8.40 (s, 2H), 8.08 (d, *J* = 8.1 Hz, 2H), 7.73 (s, 2H), 7.65 (s, 1H), 7.44 (dd, *J* = 8.2, 2.4 Hz, 4H), 7.26 – 7.07 (m, 8H), 6.64 (td, *J* = 7.4, 1.5 Hz, 2H), 6.41 (d, *J* = 2.1 Hz, 2H), 6.08 (dd, *J* = 7.6, 1.5 Hz, 2H), 5.96 (t, *J* = 2.1 Hz, 1H), 5.75 – 5.64 (m, 2H), 4.72 – 4.60 (m, 2H), 4.60 – 4.50 (m, 2H), 4.37 – 4.27 (m, 2H), 4.15 – 4.02 (m, 2H), 3.91 – 3.78 (m, 2H), 3.31 (dd, *J* = 9.9, 6.6 Hz, 2H), 3.17 (s, 6H), 3.00 – 2.86 (m, 4H), 2.59 – 2.44 (m, 2H).

^{13}C NMR spectrum (101 MHz, CD_3CN) δ 162.39, 160.96, 152.29, 145.10, 142.33, 138.57, 136.50, 131.63, 130.85, 130.38, 130.21, 128.96, 128.30, 127.19, 126.55, 125.85, 124.69, 121.98, 121.23, 112.56, 104.05, 100.08, 71.14, 71.06, 70.82, 69.43, 60.96, 55.50, 53.16, 45.01.

HRMS (ESI): cald. for $C_{58}H_{56}F_6N_5O_7P$ $[M–PF_6]^+$ *m/z* 934.4130, found 934.4141.

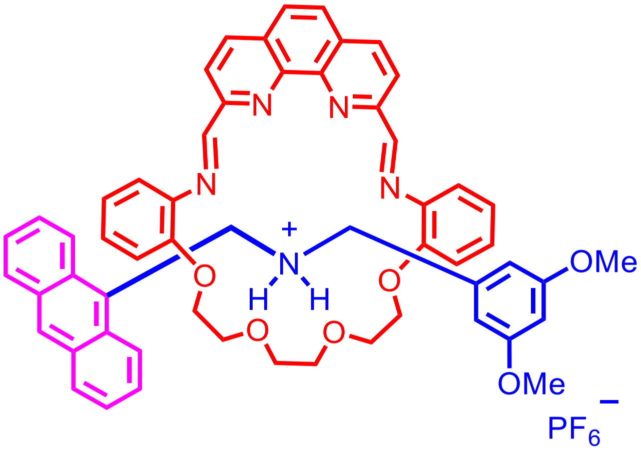

Synthesis of **9**-H·PF_6:

A solution of thread **5**-H·PF_6 (50.3 mg, 0.1 mmol) in MeCN (15 mL) was added diamine **4** (33.2 mg, 0.1 mmol) and dialdehyde **1** (23.6 mg, 0.1 mmol). The yellow mixture was stirred for overnight at room temperature. The solvents were evaporated under reduced pressure. The mixture was purified by neutral alumina column chromatography with (1/3, v/v) CH_2Cl_2/*n*-hexane, followed by CH_2Cl_2 to afford **9**-H·PF_6 as a pale-yellow powder (60.0 mg, 58%).

^{1}H NMR spectrum (400 MHz, CD_3CN) δ 10.97 (br, 2H), 8.66 (d, *J* = 8.1 Hz, 2H), 8.44 (s, 2H), 8.08 (d, *J* = 8.2 Hz, 2H), 7.69 (s, 2H), 7.64 (s, 1H), 7.52 – 7.41 (m, 4H), 7.29 – 7.24 (m, 2H), 7.22 – 7.06 (m, 6H), 6.81 (td, *J* = 7.5, 1.2 Hz, 2H), 6.42 (dd, *J* = 7.6, 1.6 Hz, 2H), 6.39 (d, *J* = 2.2 Hz, 2H), 5.95 (t, *J* = 2.2 Hz, 1H), 5.92 – 5.82 (m, 2H), 5.00 – 4.91 (m, 2H), 4.65 (t, *J* = 9.7 Hz, 2H), 4.33 (dd, *J* = 11.2, 2.6 Hz, 2H), 4.21 – 4.11 (m, 2H), 3.82 (d, *J* = 2.4 Hz, 1H), 3.80 – 3.72 (m, 3H), 3.46 – 3.35 (m, 2H), 3.17 (s, 6H).

^{13}C NMR spectrum (101 MHz, CD_3CN) δ 162.99, 161.01, 152.66, 152.35, 145.14, 142.42, 138.66, 137.28, 131.83, 130.78, 130.59, 130.00, 129.05, 128.95, 128.41, 127.25, 126.19, 125.71, 124.61, 122.26, 121.47, 112.63, 104.32, 100.23, 71.79, 70.60, 69.13, 60.96, 55.49, 52.65, 44.58.

HRMS (ESI): cald. for $C_{56}H_{52}F_6N_5O_6P$ $[M–PF_6]^+$ *m/z* 890.3918, found 890.3907.

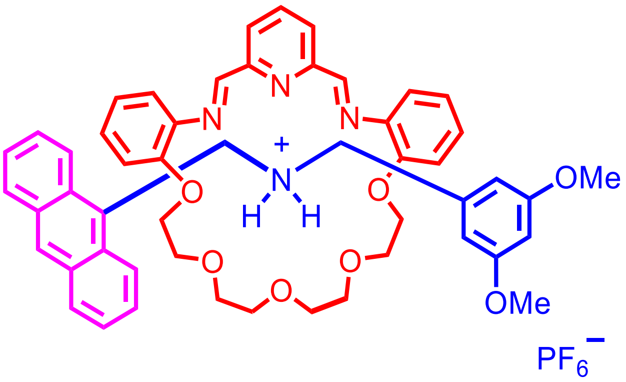

Synthesis of **10**-H·PF_6:

The synthesis of **10**-H·PF_6 was reported in the literature [3].

^{1}H NMR spectrum (400 MHz, CD_3CN) of **10**-H·PF_6. δ 9.91 (br, 2H), 8.55 (dd, J = 8.9, 1.0 Hz, 2H), 8.35 (s, 1H), 7.92 (s, 2H), 7.80 (d, J = 8.4 Hz, 2H), 7.71 (t, J = 7.7 Hz, 1H), 7.34 – 7.21 (m, 4H), 7.19 – 7.11 (m, 2H), 7.08 – 6.98 (m, 4H), 6.83 (td, J = 7.6, 1.2 Hz, 2H), 6.52 (d, J = 2.3 Hz, 2H), 6.48 (dd, J = 7.8, 1.6 Hz, 2H), 6.11 (t, J = 2.3 Hz, 1H), 5.82 (t, J = 6.6 Hz, 2H), 5.13 (t, J = 6.8 Hz, 2H), 4.35 – 4.25 (m, 4H), 4.15 – 3.99 (m, 2H), 3.87 – 3.57 (m, 10H), 3.32 (s, 6H).

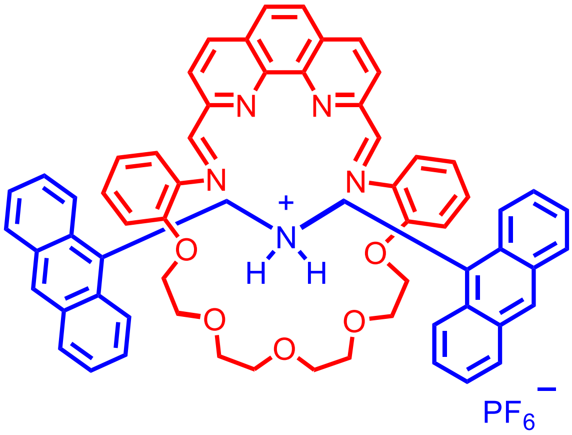

Synthesis of **11**-H·PF_6:

A solution of thread **6**-H·PF_6 (54.3 mg, 0.1 mmol) in MeCN (15 mL) was added diamine **3** (37.6 mg, 0.1 mmol) and dialdehyde **1** (23.6 mg, 0.1 mmol). The yellow mixture was stirred for overnight at room temperature. The solvents were evaporated under reduced pressure. The mixture was purified by neutral aluminum oxide column chromatography with (1/3, v/v) CH_2Cl_2/*n*-hexane, followed by CH_2Cl_2 to afford **11**-H·PF_6 as a pale-yellow powder (20.0 mg, 18%).

^{1}H NMR (400 MHz, CD_3CN) δ 10.77 (br, 2H), 8.67 (d, *J* = 8.7 Hz, 4H), 8.34 (s, 2H), 8.06 (d, *J* = 8.2 Hz, 2H), 7.87 (s, 2H), 7.72 (s, 2H), 7.66 – 7.61 (m, 4H), 7.45 (d, *J* = 8.2 Hz, 2H), 7.32 – 7.19 (m, 8H), 6.99 – 6.93 (m, 2H), 6.61 (td, *J* = 7.6, 1.1 Hz, 2H), 6.51 (dd, *J* = 8.4, 1.0 Hz, 2H), 6.17 (t, *J* = 5.7 Hz, 4H), 6.10 (dd, *J* = 7.6, 1.7 Hz, 2H), 3.94 – 3.87 (m, 4H), 3.87 – 3.82 (m, 4H), 3.38 – 3.33 (m, 4H), 3.31 (m, 4H).

^{13}C NMR (101 MHz, CD_3CN) δ 163.80, 152.56, 151.34, 145.28, 141.18, 138.59, 131.80, 131.29, 130.97, 130.63, 129.78, 128.54, 128.22, 127.74, 126.85, 125.83, 125.76, 124.79, 121.90, 112.47, 71.79, 71.73, 71.14, 68.84, 48.64.

HRMS (ESI): cald. for $C_{64}H_{56}F_6N_5O_5P$ [M–PF_6]$^+$ *m/z* 974.4281, found 974.4239.

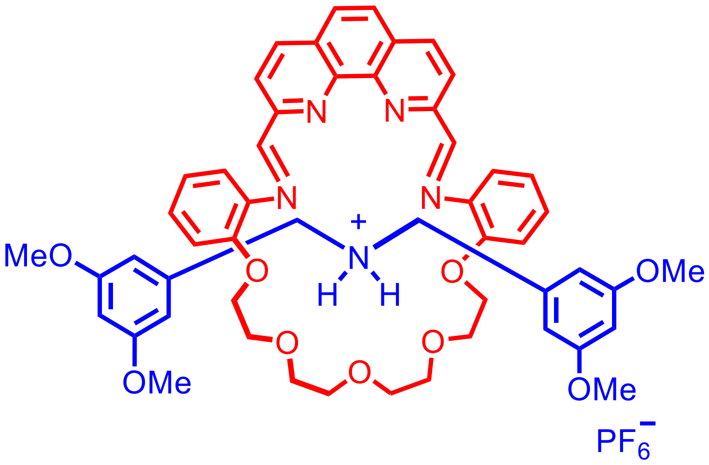

Synthesis of **12**-H·PF_6:

A solution of thread **7**-H·PF_6 (46.3 mg, 0.1 mmol) in MeCN (15 mL) was added diamine **3** (37.6 mg, 0.1 mmol) and dialdehyde **1** (23.6 mg, 0.1 mmol). The yellow mixture was stirred for overnight at room temperature. The solvents were evaporated under reduced pressure. The mixture was purified by neutral aluminum oxide column chromatography with (1/3, v/v) CH_2Cl_2/*n*-hexane, followed by CH_2Cl_2 to afford **12**-H·PF_6 as a pale-yellow powder (63.0 mg, 61%).

^{1}H NMR (400 MHz, CD_3CN) δ 10.84 (br, 2H), 8.68 (s, 2H), 8.58 (d, *J* = 8.2 Hz, 2H), 8.10 (d, *J* = 8.2 Hz, 2H), 8.01 (s, 2H), 7.26 – 7.17 (m, 2H), 7.12 (dd, *J* = 8.4, 1.2 Hz, 2H), 6.77 (td, *J* = 7.6, 1.2 Hz, 2H), 6.34 (dd, *J* = 7.7, 1.6 Hz, 2H), 6.17 (d, *J* = 2.3 Hz, 4H), 5.75 (t, *J* = 2.2 Hz, 2H), 4.38 – 4.25 (m, 8H), 3.86 – 3.76 (m, 4H), 3.38 – 3.27 (m, 4H), 3.20 (s, 12H), 3.10 – 3.03 (m, 4H).

^{13}C NMR (101 MHz, CD_3CN) δ 163.37, 160.62, 152.46, 151.99, 146.51, 142.25, 139.14, 135.65, 131.48, 129.00, 128.62, 127.18, 122.19, 121.53, 113.58, 106.86, 100.09, 71.59, 71.02, 71.00, 69.73, 55.37, 52.87.

HRMS (ESI): cald. for $C_{52}H_{56}F_6N_5O_9P$ [M–PF_6]$^+$ *m/z* 894.4078, found 894.4038.

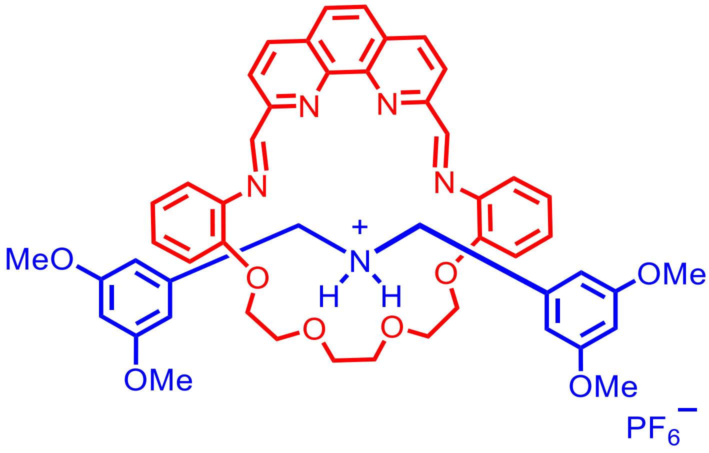

Synthesis of **13**-H·PF_6:

A solution of thread **7**-H·PF_6 (46.3 mg, 0.1 mmol) in MeCN (15 mL) was added diamine **4** (33.2 mg, 0.1 mmol) and dialdehyde **1** (23.6 mg, 0.1 mmol). The yellow mixture was stirred for overnight at room temperature. The solvents were evaporated under reduced pressure. The mixture was purified by recrystallization in MeCN/Et_2O solution. **13**-H·PF_6 as a pale-yellow powder was obtained (50.0 mg, 50%).

^{1}H NMR (400 MHz, CD_3CN) δ 10.93 (br, 2H), 8.69 (s, 2H), 8.57 (d, *J* = 8.2 Hz, 2H), 8.10 (d, *J* = 8.2 Hz, 2H), 8.00 (s, 2H), 7.32 – 7.23 (m, 2H), 7.14 (d, *J* = 7.7 Hz, 2H), 6.89 (td, *J* = 7.6, 1.0 Hz, 2H), 6.59 (dd, *J* = 7.6, 1.5 Hz, 2H), 6.18 (d, *J* = 2.2 Hz, 4H), 5.74 (t, *J* = 2.2 Hz, 2H), 4.61 – 4.48 (m, 4H), 4.37 – 4.26 (m, 4H), 3.84 – 3.77 (m, 4H), 3.44 (s, 4H), 3.19 (s, 12H).

^{13}C NMR (101 MHz, CD_3CN) δ 163.52, 160.84, 152.55, 146.60, 142.41, 139.29, 136.29, 131.68, 129.15, 127.79, 122.37, 121.63, 113.27, 107.08, 100.16, 71.45, 70.64, 69.54, 55.43, 52.57.

HRMS (ESI): cald. for $C_{50}H_{52}F_6N_5O_8P$ $[M–PF_6]^+$ *m/z* 850.3816, found 850.3779.

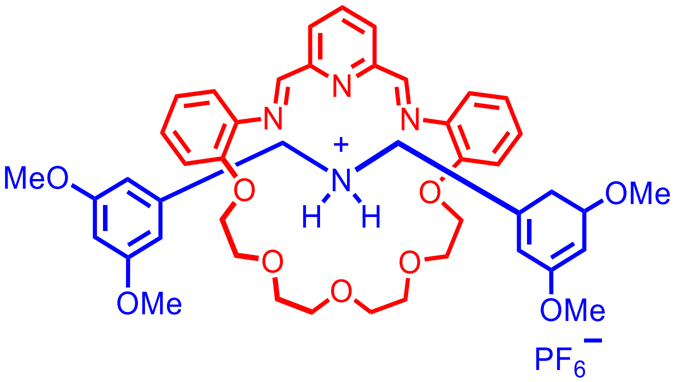

Synthesis of **14**-H·PF_6:

The synthesis of **14**-H·PF_6 was reported in the literature [5].

Table S1. NMR chemical shift value (δ in ppm) of the benzylic protons of the threads and the rotaxanes[a]

Compounds	chemical shift value of benzylic protons (anthracene)	chemical shift value of benzylic protons (3,5-dimethoxybenzyl)
Thread **5**-H·PF_6	5.24	4.41
Rotaxane **8**-H·PF_6	5.75 – 5.64	4.72 – 4.60
Rotaxane **9**-H·PF_6	5.92 – 5.82	5.00 – 4.91
Rotaxane **10**-H·PF_6	5.87 – 5.79	5.16 – 5.09
Thread **6**-H·PF_6	5.25	
Rotaxane **11**-H·PF_6	6.20 – 6.14	
Thread **7**-H·PF_6		4.12[6]
Rotaxane **12**-H·PF_6		4.32 – 4.28
Rotaxane **13**-H·PF_6		4.60 – 4.52
Rotaxane **14**-H·PF_6		4.62 – 4.53

[a] Value obtained from ^{1}H NMR spectroscopy (400 MHz, CD_3CN, 298 K)

NMR spectra

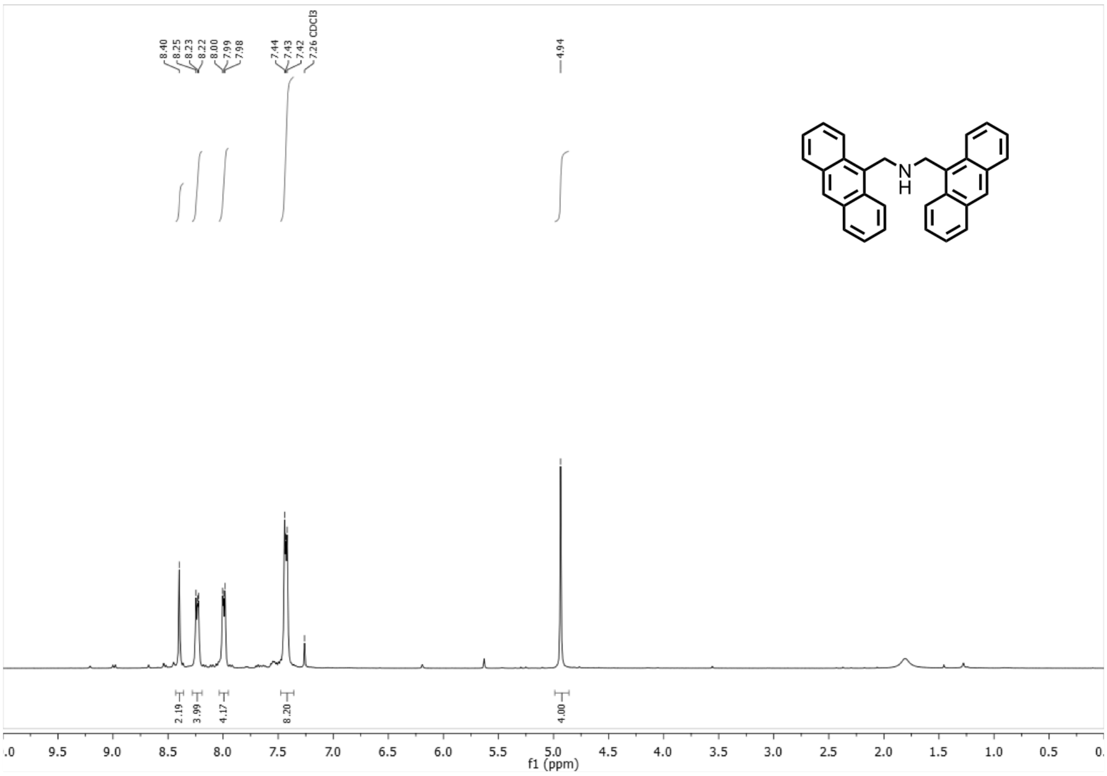

Figure S1. ^{1}H NMR spectrum (400 MHz, $CDCl_3$) of **6**.

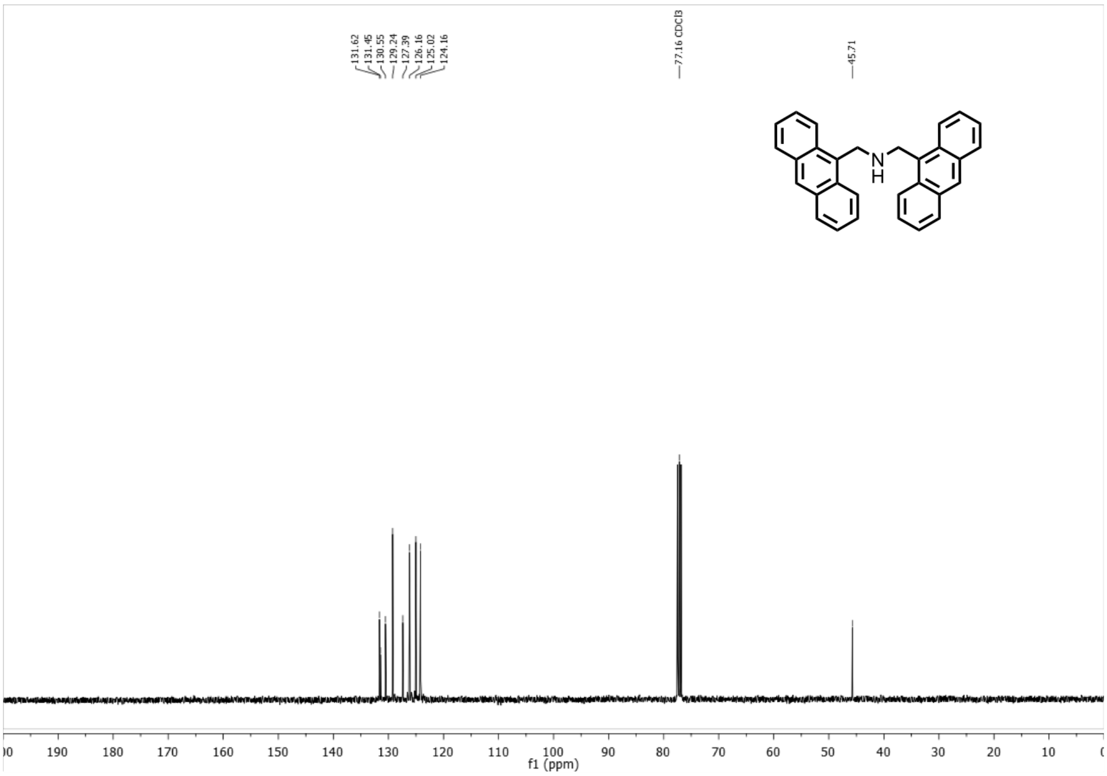

Figure S2. ^{13}C NMR spectrum (101 MHz, $CDCl_3$) of **6**.

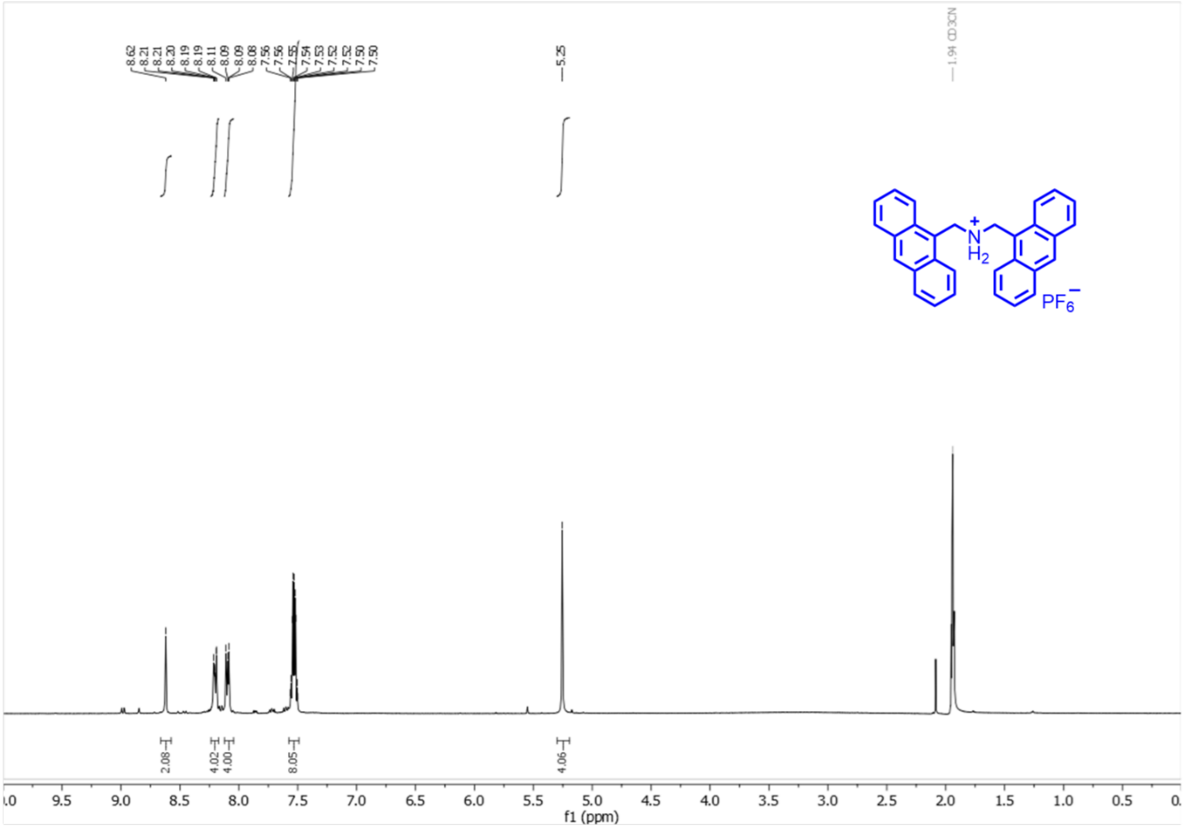

Figure S3. ^{1}H NMR spectrum (400 MHz, CD_3CN) of **6**-H·PF_6.

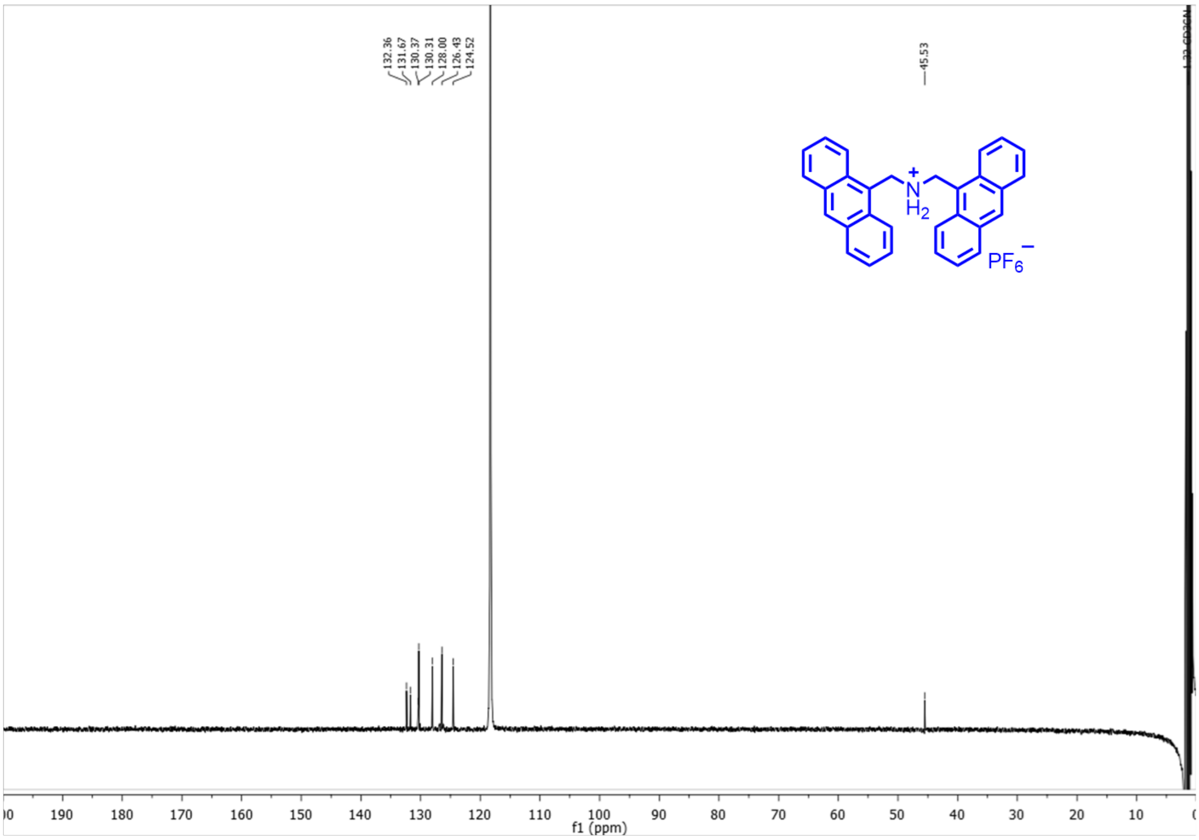

Figure S4. ^{13}C NMR spectrum (101 MHz, CD_3CN) of **6**-H·PF_6.

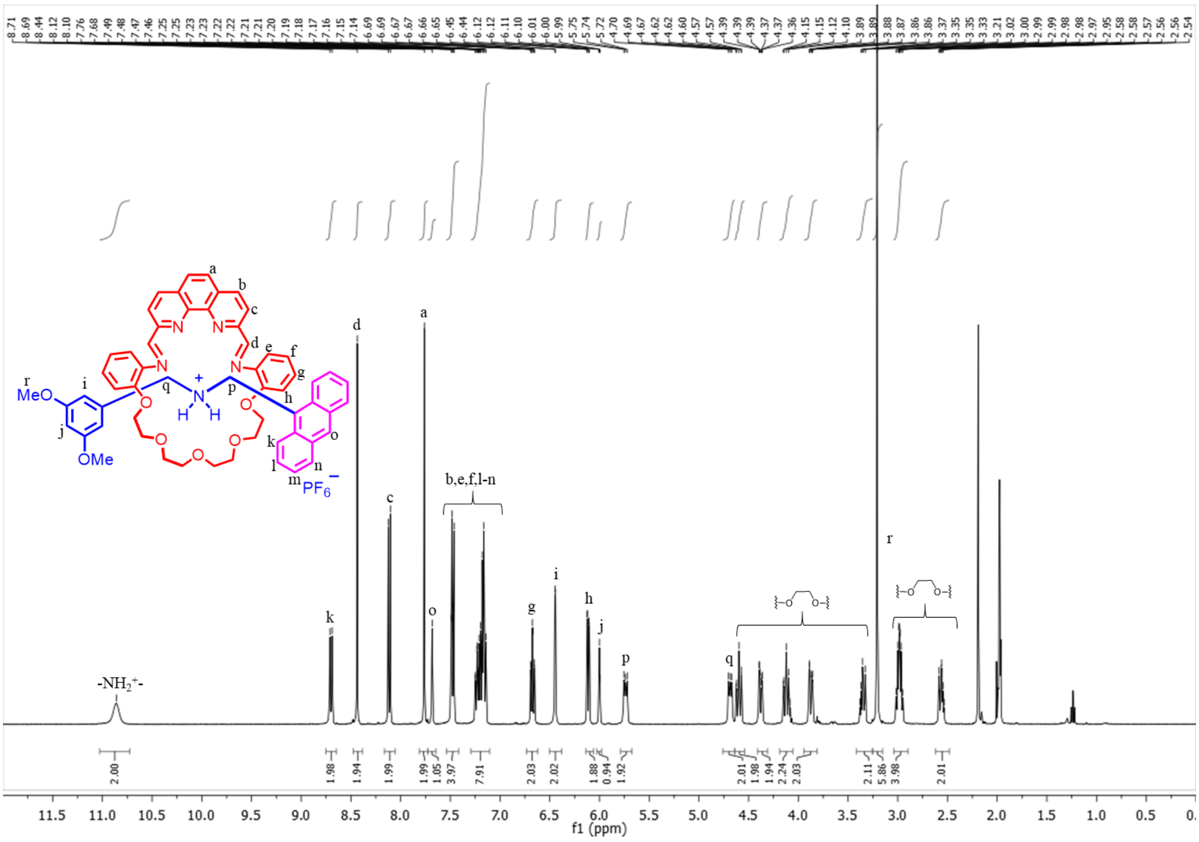

Figure S5. ^{1}H NMR spectrum (400 MHz, CD_3CN) of **8**-H·PF_6.

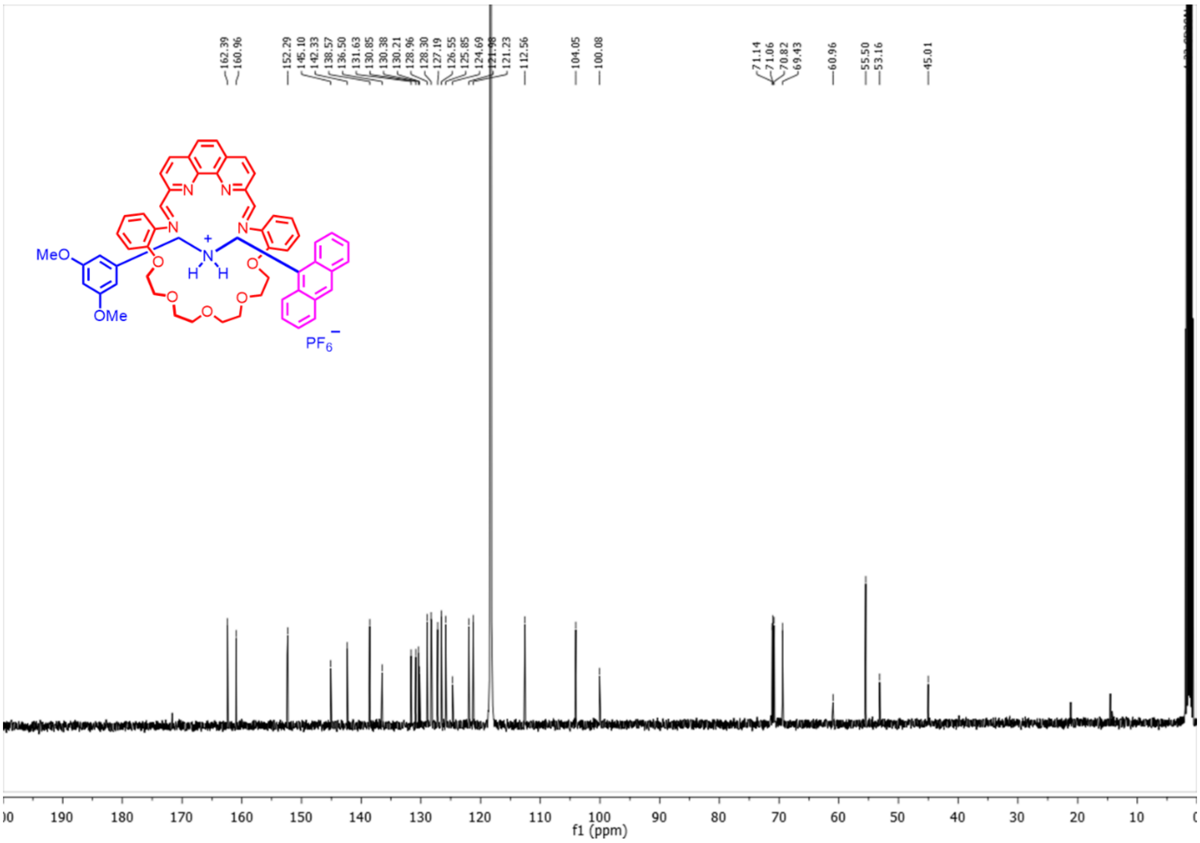

Figure S6. ^{13}C NMR spectrum (101 MHz, CD_3CN) of **8**-H·PF_6.

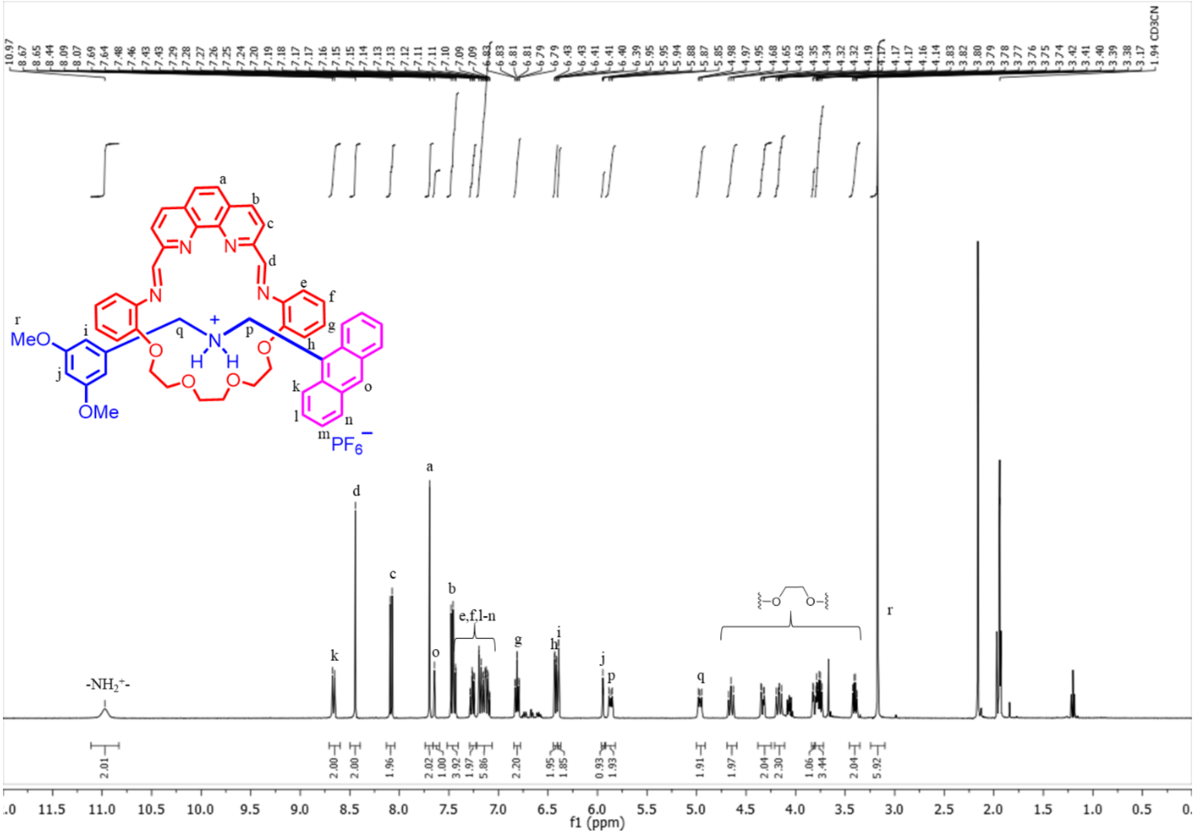

Figure S7. ^{1}H NMR spectrum (400 MHz, CD_3CN) of **9**-H·PF_6.

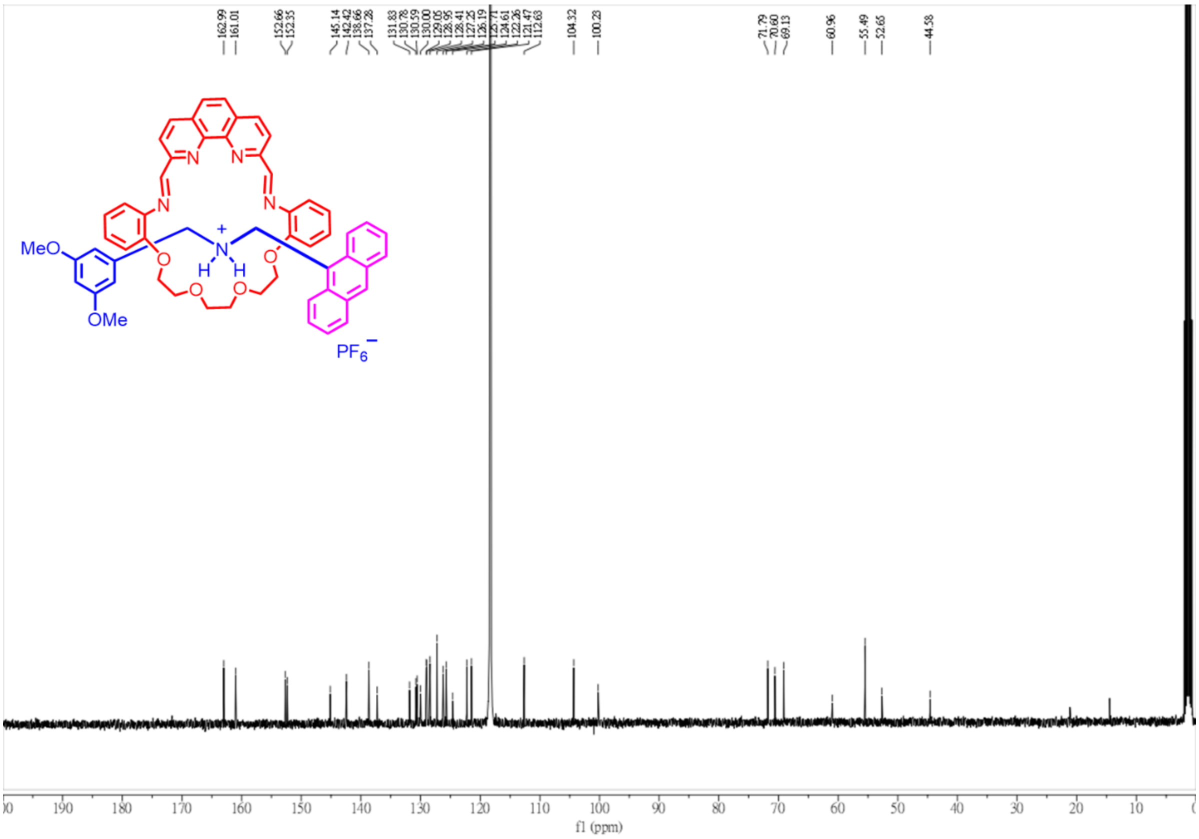

Figure S8. ^{13}C NMR spectrum (101 MHz, CD_3CN) of **9**-H·PF_6.

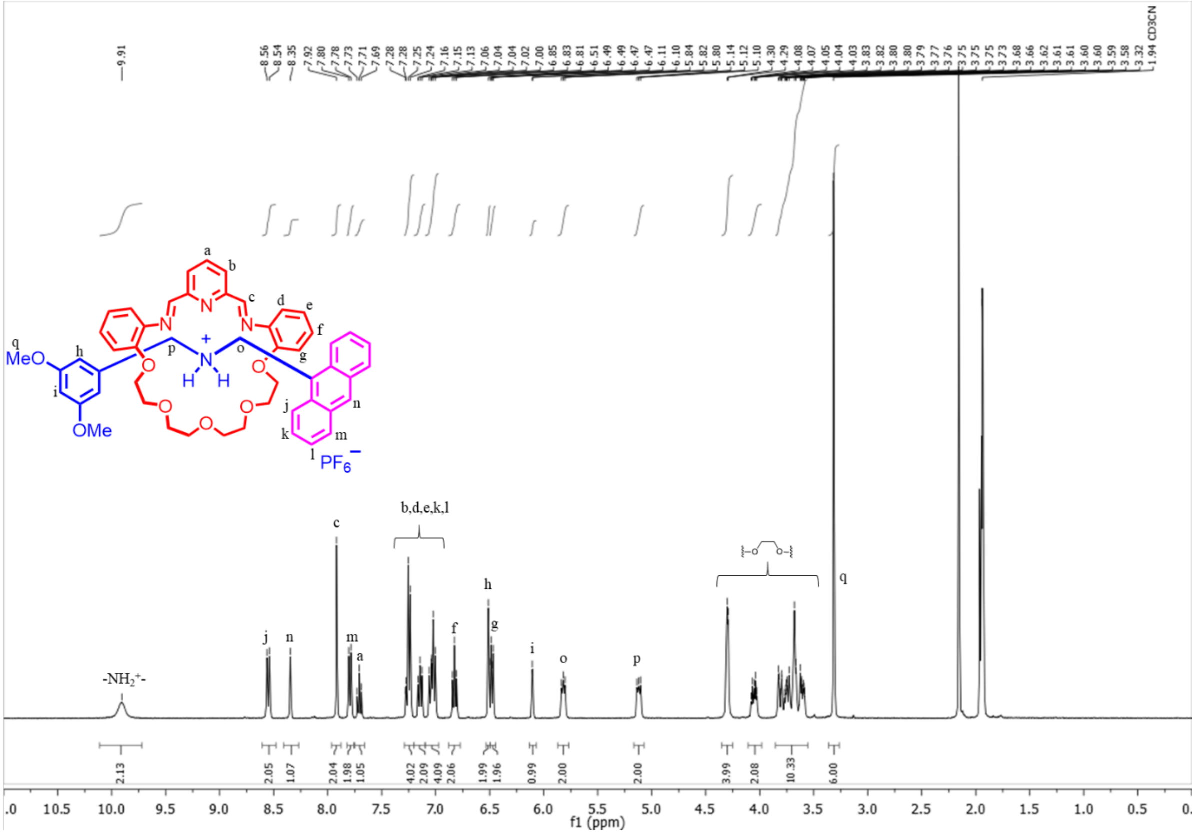

Figure S9. ^{1}H NMR spectrum (400 MHz, CD_3CN) of **10**-H·PF_6.

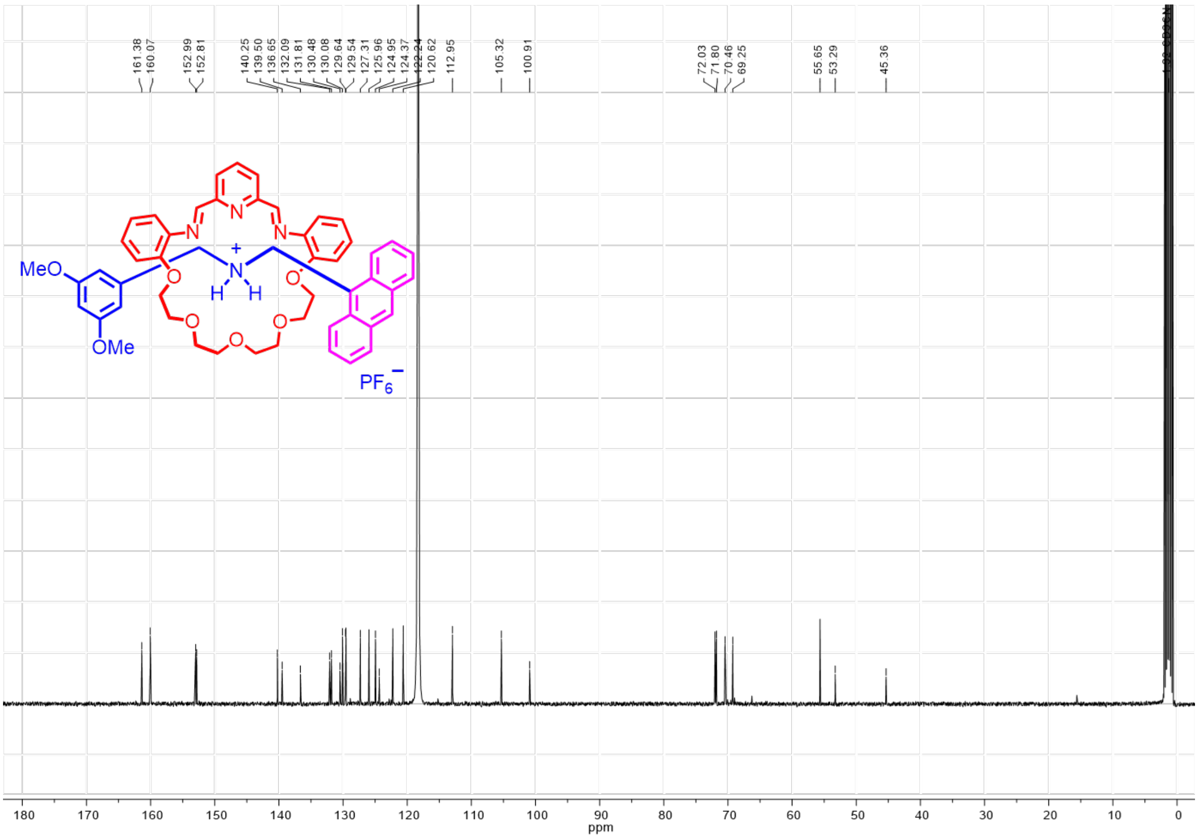

Figure S10. ^{13}C NMR spectrum (101 MHz, CD_3CN) of **10**-H·PF_6.

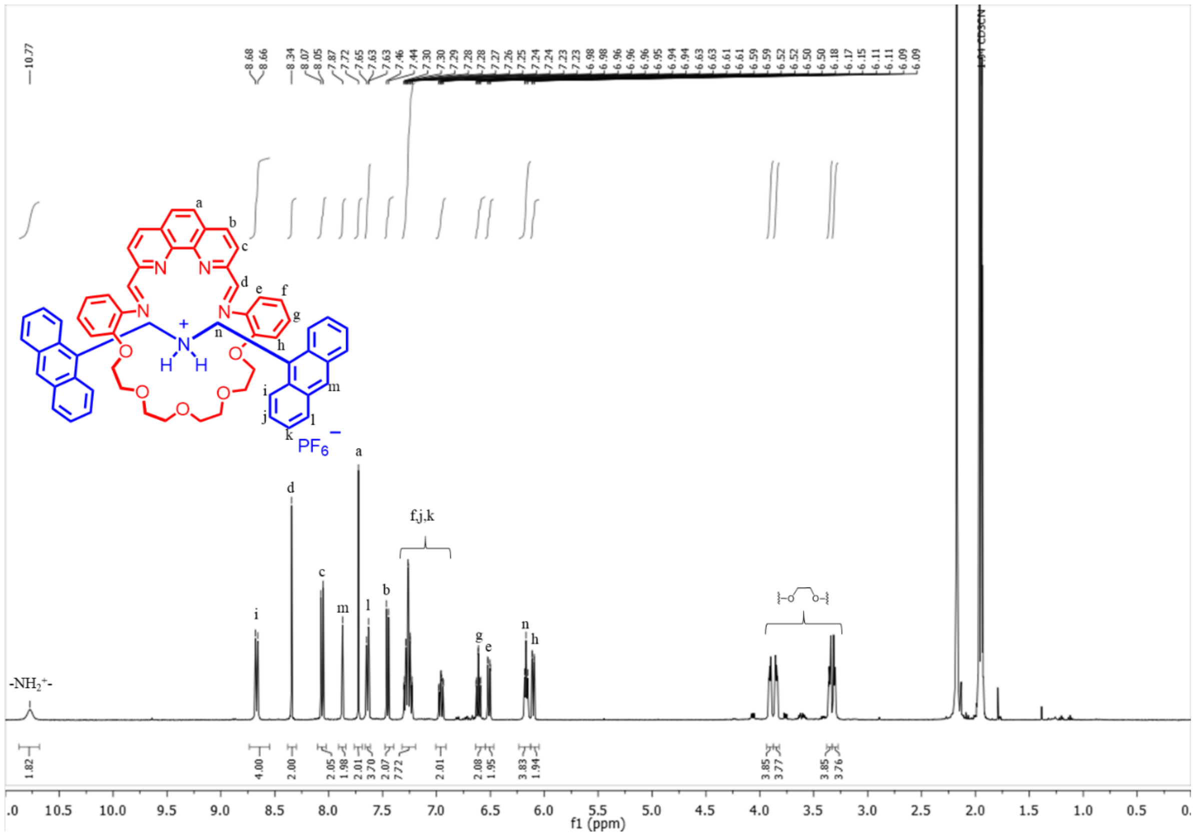

Figure S11. ^{1}H NMR spectrum (400 MHz, CD_3CN) of **11**-H·PF_6.

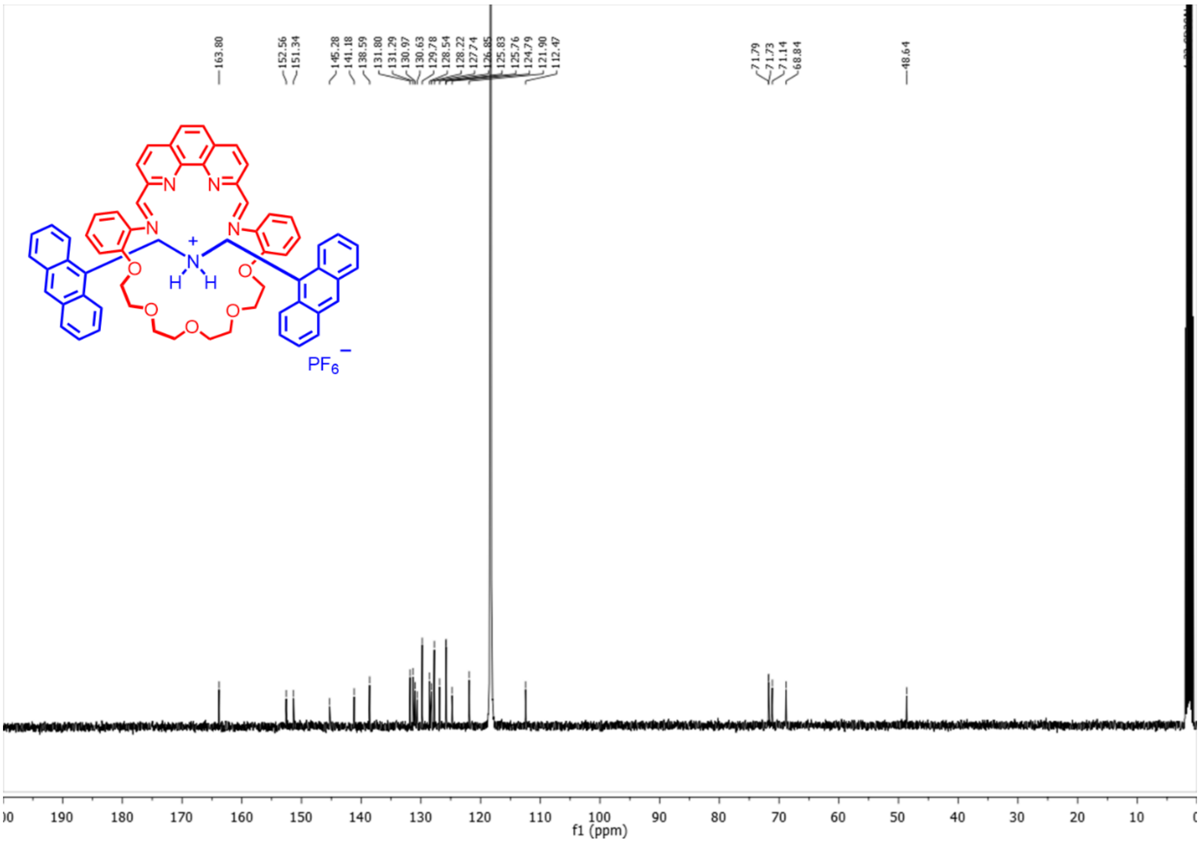

Figure S12. ^{13}C NMR spectrum (101 MHz, CD_3CN) of **11**-H·PF_6.

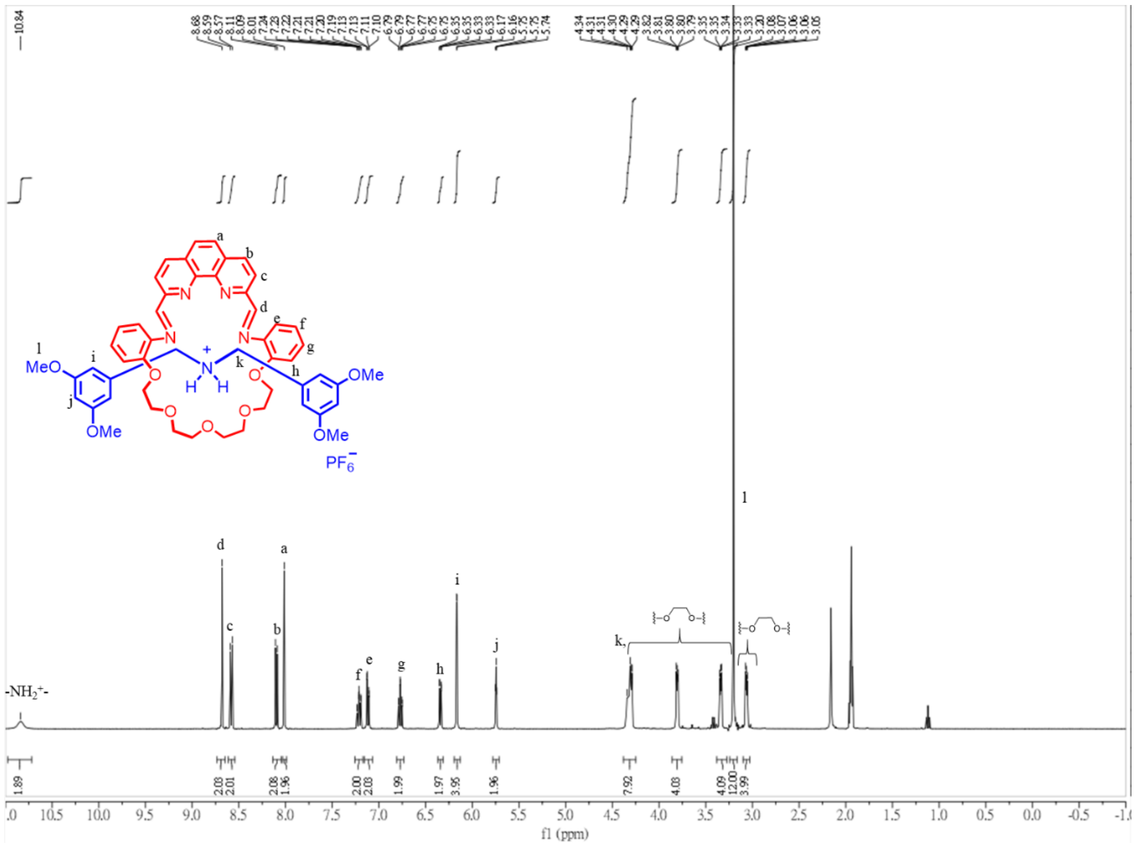

Figure S13. ^{1}H NMR spectrum (400 MHz, CD_3CN) of **12**-H·PF_6.

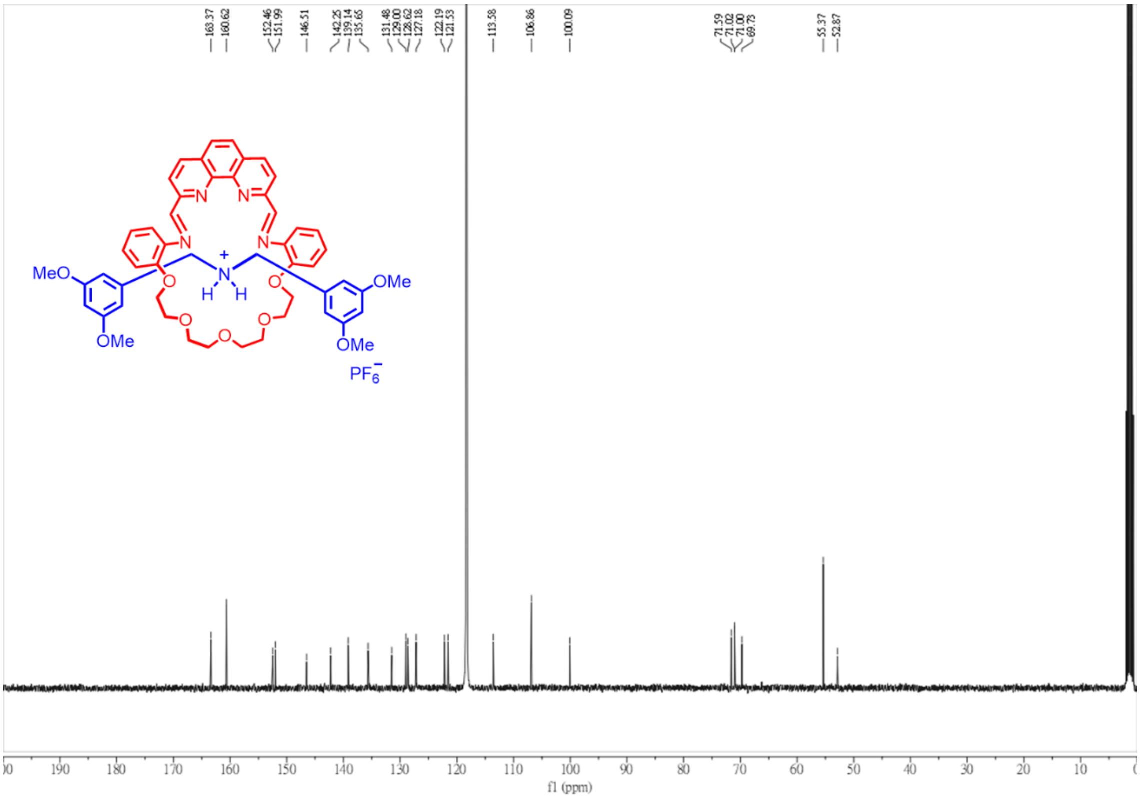

Figure S14. ^{13}C NMR spectrum (101 MHz, CD_3CN) of **12**-H·PF_6.

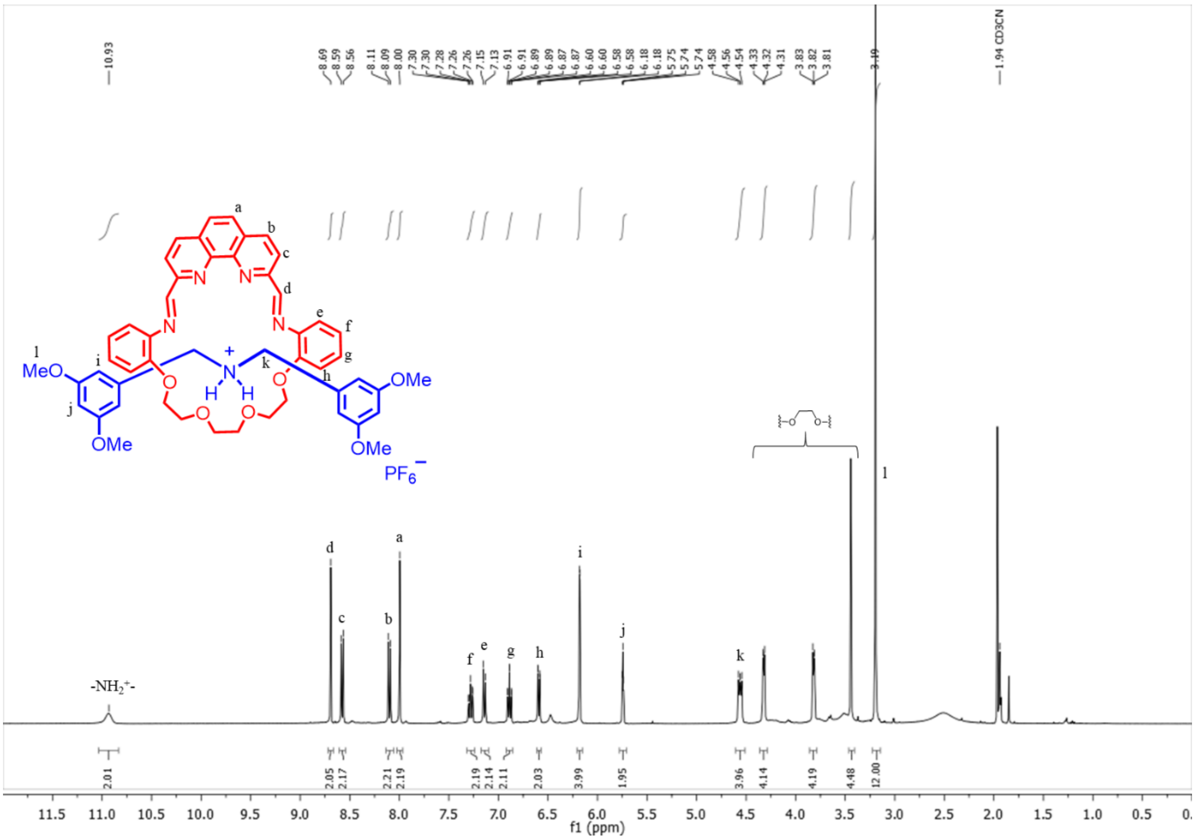

Figure S15. ^{1}H NMR spectrum (400 MHz, CD_3CN) of **13**-H·PF_6.

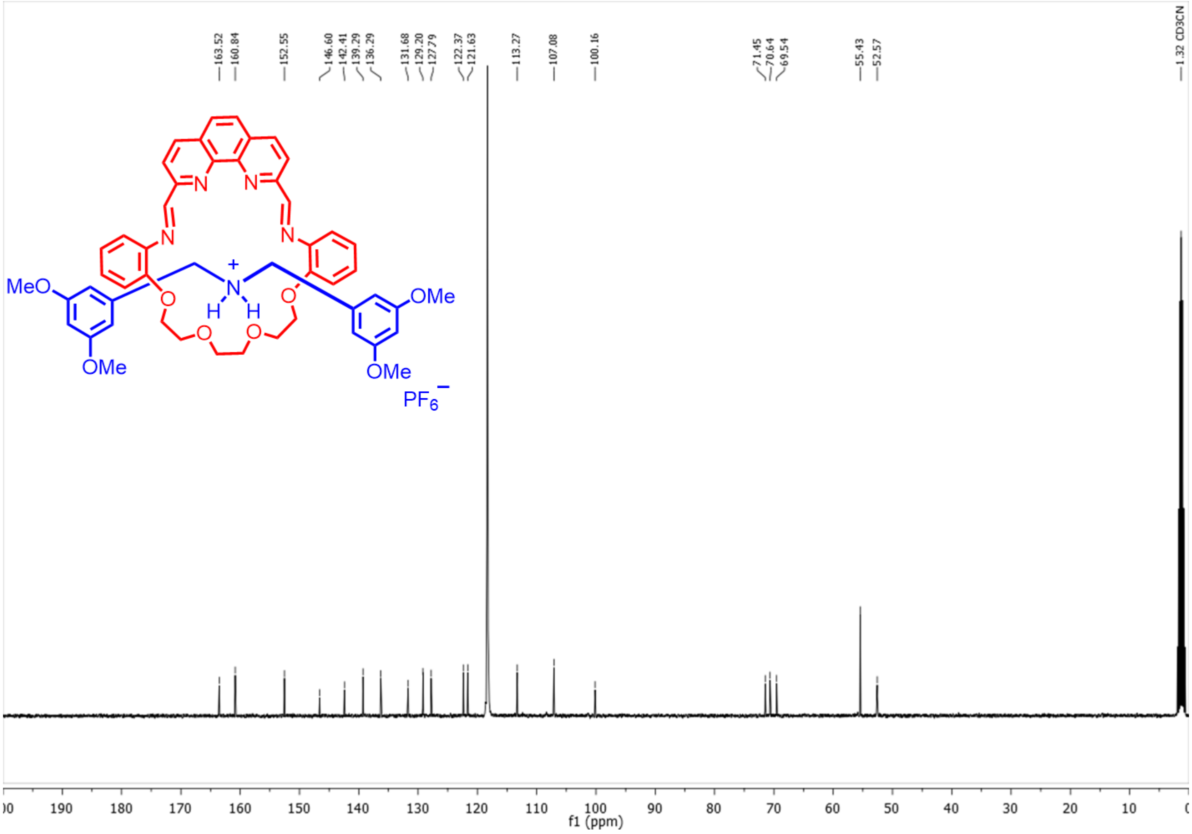

Figure S16. ^{13}C NMR spectrum (101 MHz, CD_3CN) of **13**-H·PF_6.

Stacked ^{1}H NMR spectra for the DCL experiments

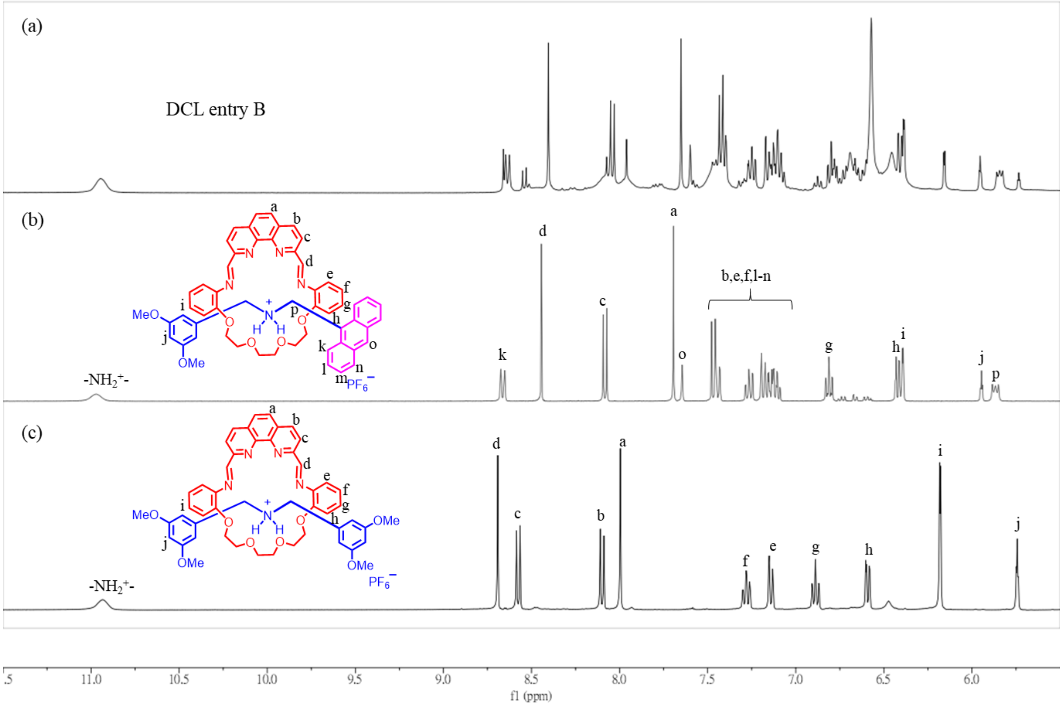

Figure S17. Partial Stacked ^{1}H NMR spectra (CD_3CN, 400 MHz) of (a) crude product mixture of DCL entry B after 18 h reaction, (b) **9**-H·PF_6, (c) **13**-H·PF_6.

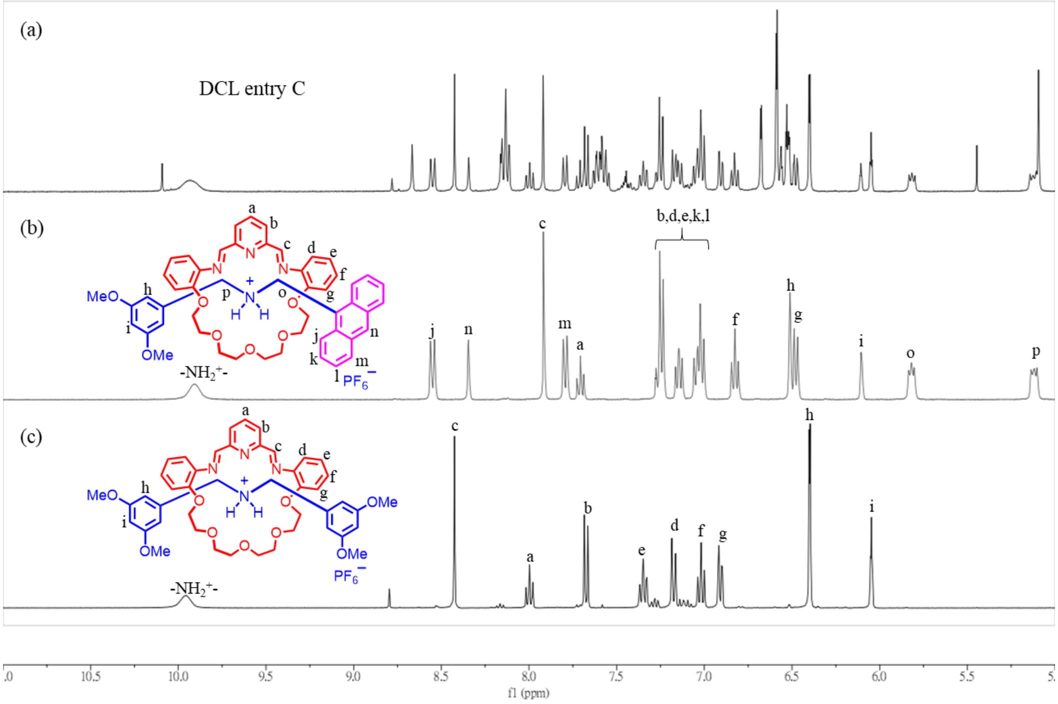

Figure S18. Partial Stacked ^{1}H NMR spectra (CD_3CN, 400 MHz) of (a) crude product mixture of DCL entry C after 18 h reaction, (b) **10**-H·PF_6, (c) **14**-H·PF_6.

Mass spectra

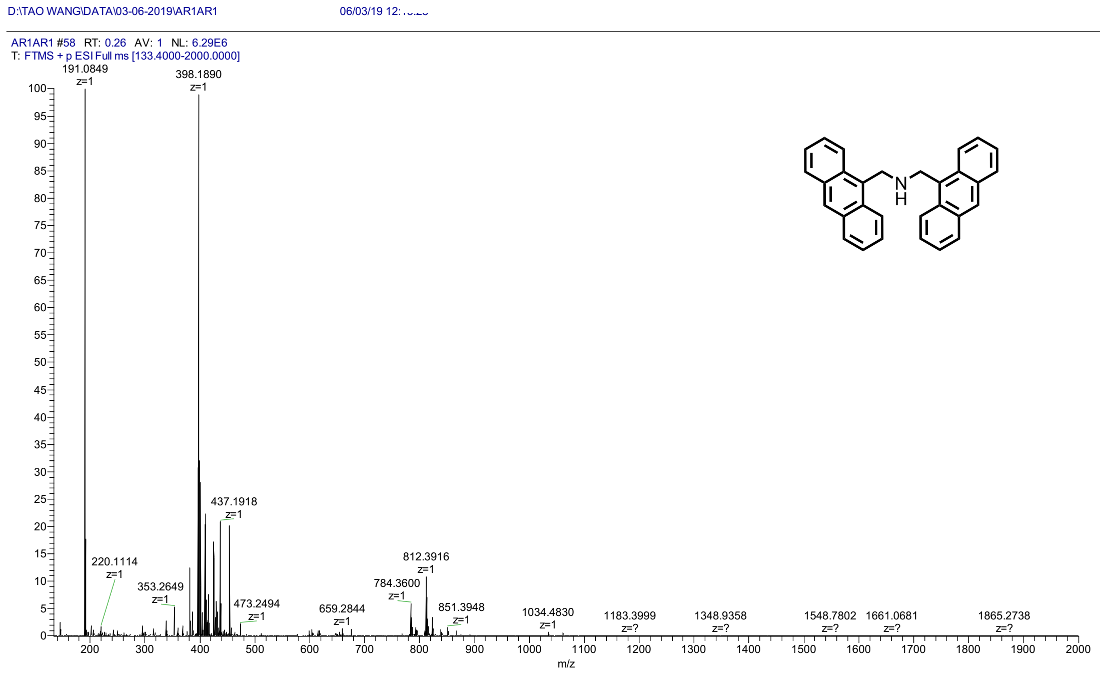

Figure S19. HRMS (ESI) of compound **6.**

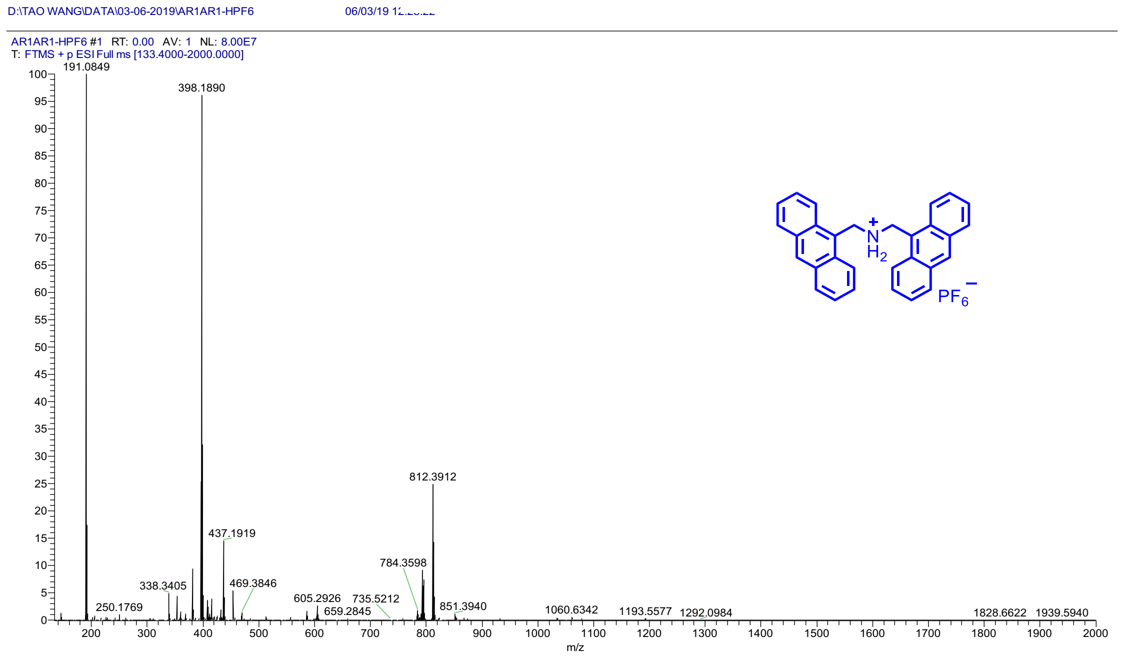

Figure S20. HRMS (ESI) of compound **6**-H·PF_6.

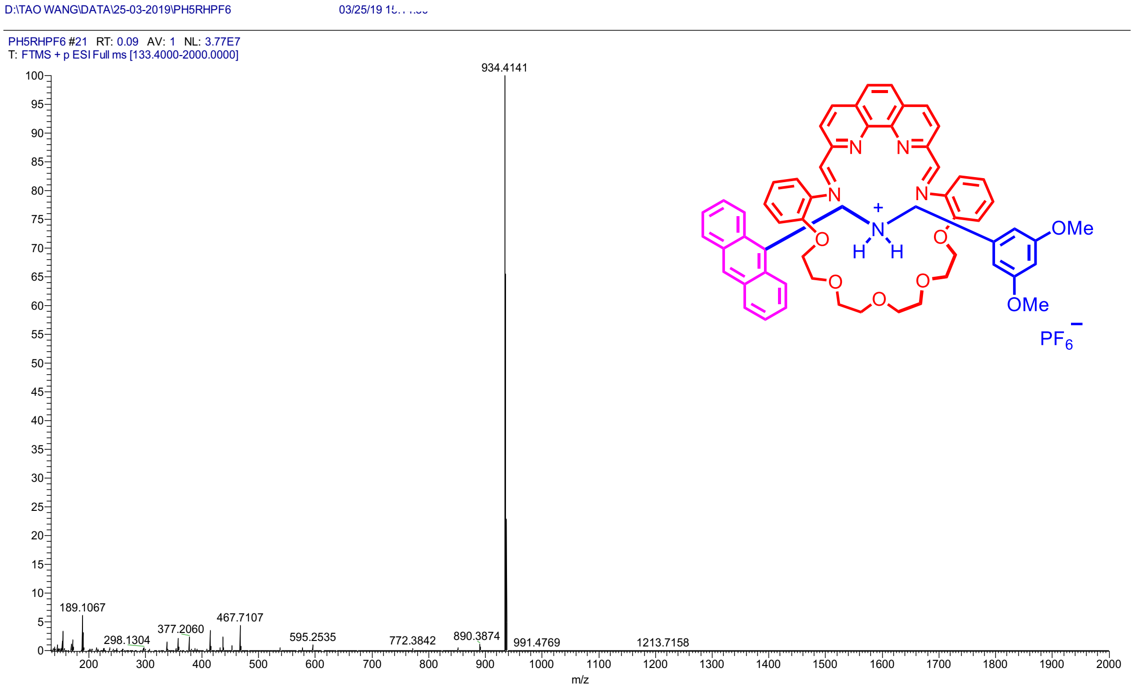

Figure S21. HRMS (ESI) of **8**-H·PF_6**.**

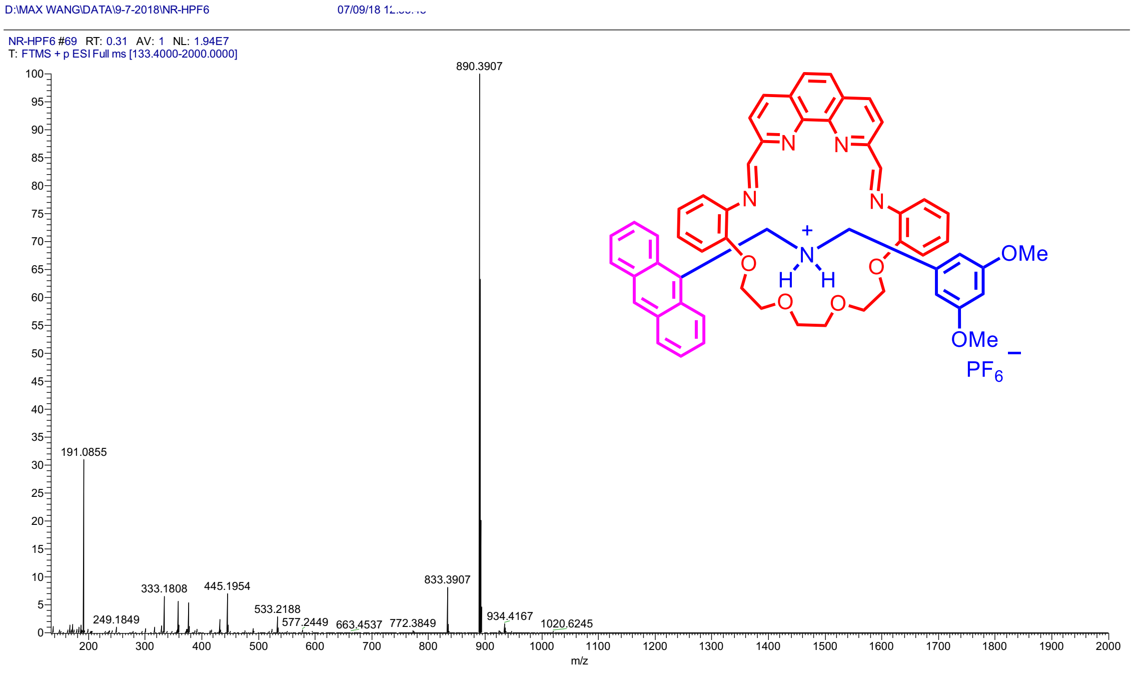

Figure S22. HRMS (ESI) of **9**-H·PF_6**.**

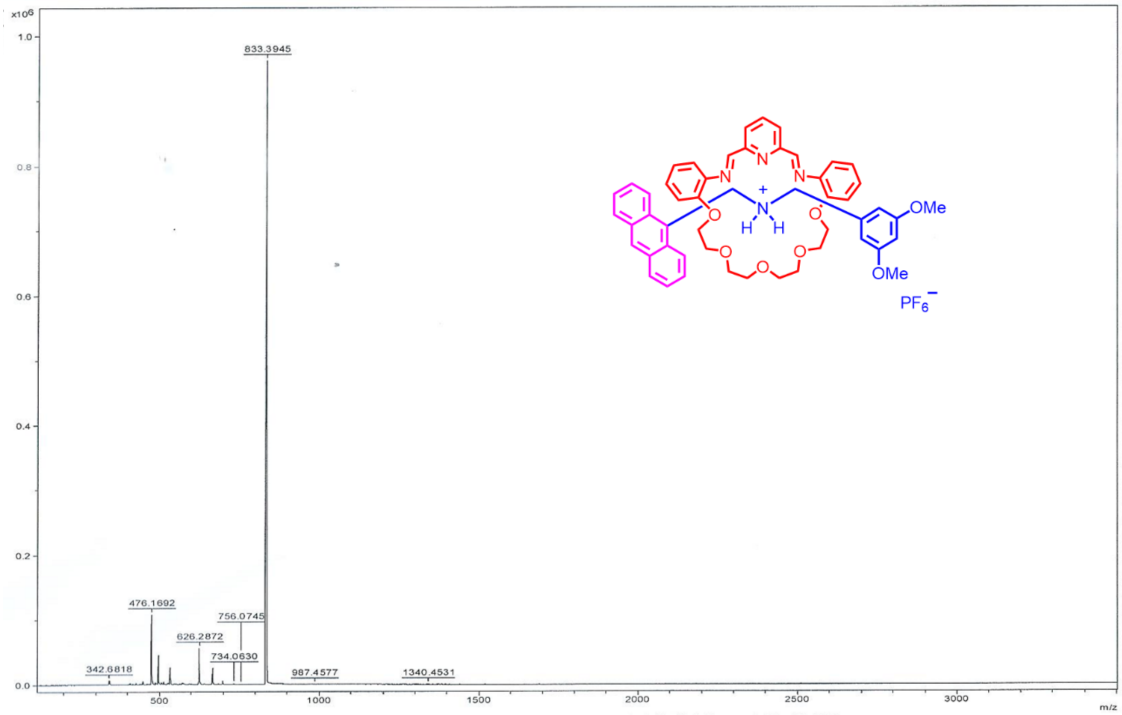

Figure S23. HRMS (MALDI-TOF) of **10**-H·PF_6**.**

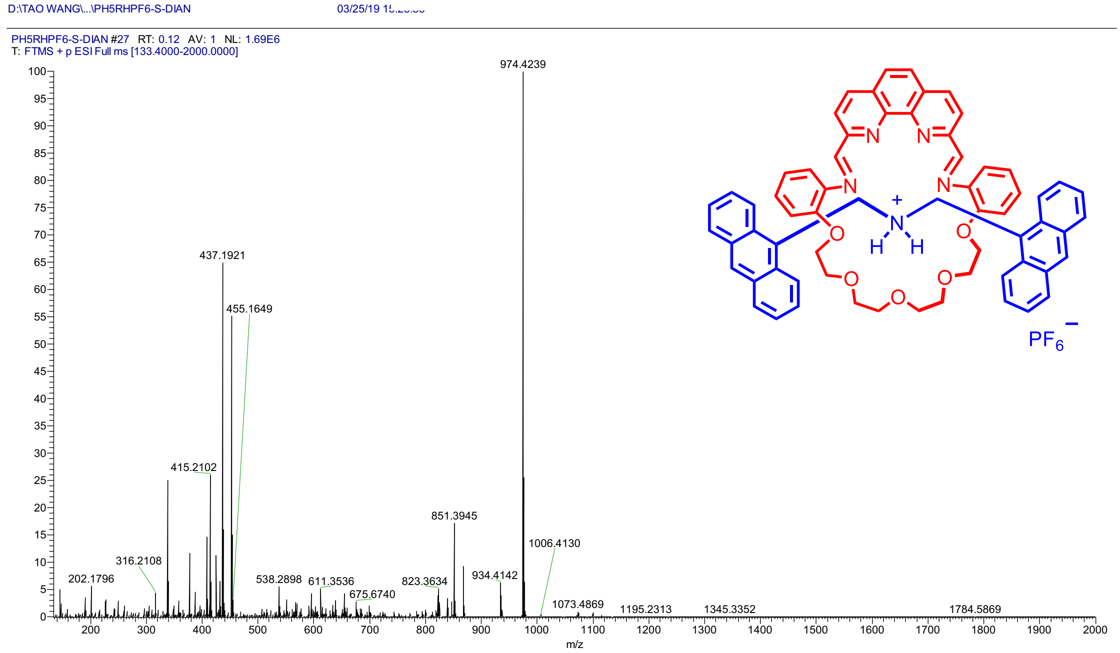

Figure S24. HRMS (ESI) of **11**-H·PF_6**.**

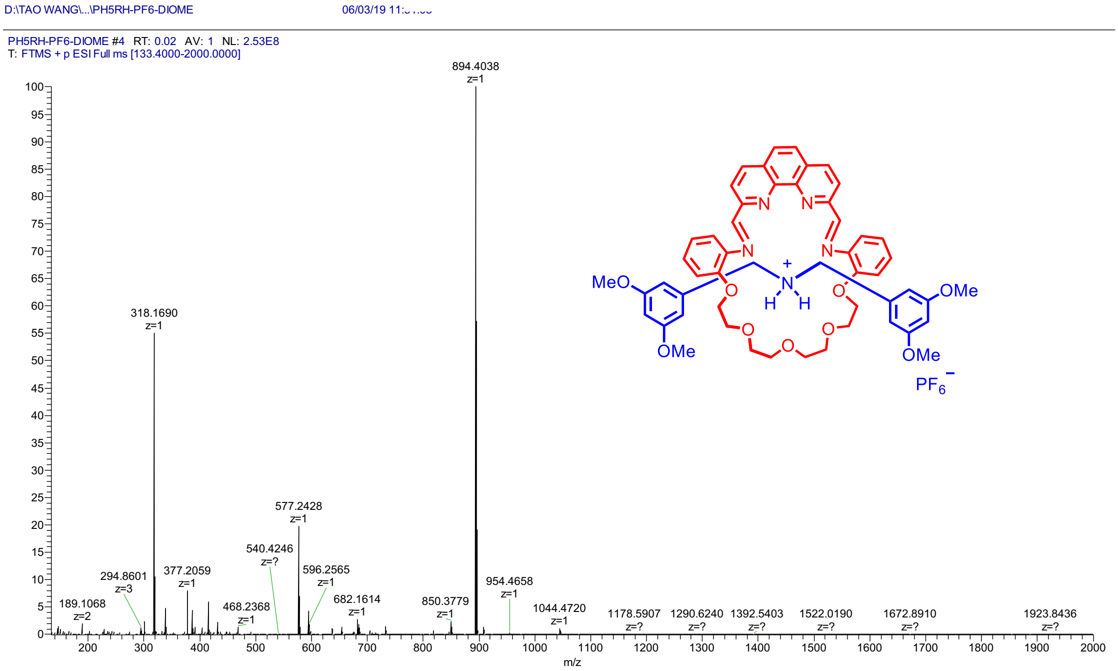

Figure S25. HRMS (ESI) of **12**-H·PF_6**.**

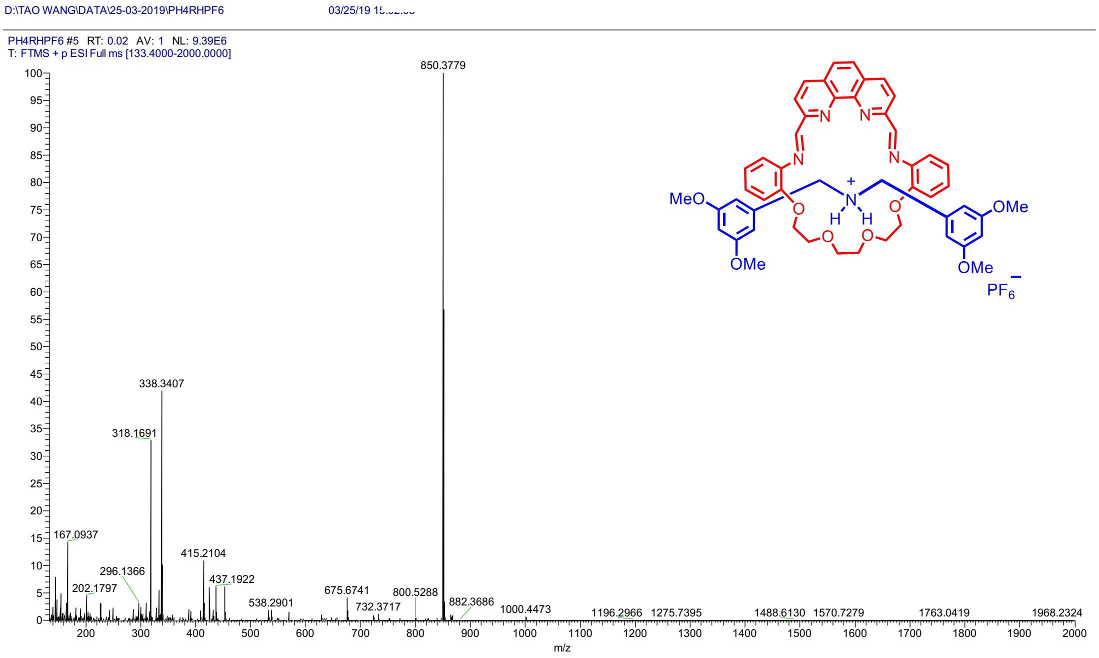

Figure S26. HRMS (ESI) of **13**-H·PF_6**.**

X-Ray crystallography

Selected crystals were used for intensity data collection on a Bruker AXS Kappa Apex II Duo diffractometer at 173 K using frames of oscillation range 0.3°, with 2° < θ < 28°. An empirical absorption correction was applied using the SADABS program [7]. The structures were solved by the direct method and refined by full-matrix least-squares on F^2 using the SHELXTL program package [8].

The crystal structures of rotaxanes **8**-H·PF_6 – **10**-H·PF_6 have been determined by single-crystal X-ray analysis. The crystal data and refinement parameters are presented in Table S2. All crystal drawings are produced by using software Diamond 3.0.

Table S2. Crystallography data and structure refinement parameters of [2]rotaxanes 8-H·PF_6 – 10-H·PF_6.

**Complex**	**8**-H·PF_6	**9**-H·PF_6	**10**-H·PF_6
**Formula**	$C_{58}H_{56}F_6N_5O_7P$	$C_{56}H_{53}F_6N_5O_6P$	$C_{110}H_{118}F_{12}N_{12}O_{14}P_2$
**Formula Wt.**	1080.04	1036.00	2122.10
**Crystal System**	Monoclinic	Monoclinic	Triclinic
**Space Group**	$P2_1/n$	C2/c	P-1
a **[Å]**	11.335(4)	28.716(16)	14.868(3)
b **[Å]**	27.667(9)	11.944(7)	17.598(4)
c **[Å]**	17.518(6)	33.560(19)	23.155(5)
α **[Å]**	90	90	69.301(4)
β **[Å]**	98.027(7)	108.951(10)	76.238(4)
γ **[Å]**	90	90	69.289(4)
**V [$Å^3$]**	5440(3)	10887(11)	5257.0(19)
**Z**	4	8	2
ρ_c**[gcm^{-3}]**	1.304	1.264	1.341
μ **[mm^{-1}]**	0.128	0.124	0.132
R_1[a] **($I>2\sigma$)**	0.0638	0.0686	0.0616
wR_2[b] **(all data)**	0.2025	0.2069	0.1861
**GOF**	1.035	1.045	1.027

Summary of validation response

*Rotaxane **8**-H•PF_6*

- A solvent void contains one to two disordered dichloromethane molecules, which is consistent with the crystallization conditions. And a solvent mask was applied for that.

- The large errors on the unit cell lengths and unit cell volume is speculated to be arose from the crystal not sufficiently be centered and the deficiencies from the used instrument.

_vrf_PLAT220
PROBLEM: NonSolvent Resd 1 C Ueq(max)/Ueq(min) Range 5.1 Ratio
RESPONSE: Due to the slightly poor data collected
_vrf_PLAT241
PROBLEM: High 'MainMol' Ueq as Compared to Neighbors of C48 Check
RESPONSE: Due to the disordered atoms of C47 and C47A
_vrf_PLAT260
PROBLEM: Large Average Ueq of Residue Including P1 0.101 Check
RESPONSE: Due to the slightly poor data collected
_vrf_PLAT329
PROBLEM: Carbon Atom Hybridisation Unclear for .... C51 Check
RESPONSE: Should be sp3; but not shown due to the disorder
_vrf_PLAT911
PROBLEM: Missing FCF Refl Between Thmin & STh/L= 0.600 3 Report
RESPONSE: Due to the slightly poor data collected
_vrf_PLAT918
PROBLEM: Reflection(s) with I(obs) much Smaller I(calc) . 3 Check
RESPONSE: Due to the use of solvent mask for two disordered dichloromethane molecules
_vrf_PLAT975
PROBLEM: Check Calcd Resid. Dens. 0.97A From C52 0.56 eA-3
RESPONSE: missed hydrogen atoms due to the disordered C51 and C51A

*Rotaxane **9**-H•PF_6*

- Large residual peaks are found around the PF_6 counter anions, but those are confirmed not to be the disorder components arose from either the P or F atoms.
- A solvent void contains two unsolvable acetonitrile molecules, which is consistent with the crystallization conditions. And a solvent mask was applied for that.
- Extreme short H•••H contact distance between H8B and H21 is considered to be driven by the steric constraints imposed by the structure, in combination with the strong π-π stacking interaction between the phenanthroline and the anthracene.
- The large errors on the unit cell lengths and unit cell volume is speculated to be arose from the crystal not sufficiently be centered and the deficiencies from the used instrument.

*Rotaxane **10**-H•PF_6*

- The large errors on the unit cell lengths and unit cell volume is speculated to be arose from the crystal not sufficiently be centered and the deficiencies from the used instrument.

_vrf_PLAT230

PROBLEM: Hirshfeld Test Diff for C41 --C42 . 9.7 s.u.

RESPONSE: the residual density next to C42 is checked and not considered as the disorder component of C42.

_vrf_PLAT360

PROBLEM: Short C(sp3)-C(sp3) Bond C88 - C89 . 1.33 Ang.

RESPONSE: Due to the large ellipsoid sizes of both C88 and C89 but no acceptable disorder components are found

_vrf_PLAT410

PROBLEM: Short Intra H...H Contact H86 ..H88A . 1.81 Ang.

RESPONSE: the steric constraint of the structure forces the orientation of C86 close to C88

References

[1] Sharghi, H.; Nasseri, M. A.; Niknam, K. Phenol-Containing Macrocyclic Diamides as New Catalysts in the Highly Regioselective Conversion of Epoxides to β-Hydroxy Thiocyanates. *J. Org. Chem.* **2001**, *66* (22), 7287–7293.

[2] Xiao, C.-L.; Wang, C.-Z.; Yuan, L.-Y.; Li, B.; He, H.; Wang, S.; Zhao, Y.-L.; Chai, Z.-F.; Shi, W.-Q. Excellent Selectivity for Actinides with a Tetradentate 2,9-Diamide-1,10-Phenanthroline Ligand in Highly Acidic Solution: A Hard–Soft Donor Combined Strategy. *Inorg. Chem.* **2014**, *53* (3), 1712–1720.

[3] Wong, W.-Y.; Leung, K. C.-F.; Stoddart, J. F. Self-assembly, Stability Quantification, Controlled Molecular Switching, and Sensing Properties of an Anthracene-containing Dynamic [2]Rotaxane. *Org. Biomol. Chem.* **2010**, *8* (10), 2332–2343.

[4] Lan, P.; Berta, D.; Porco, J. A.; South, M. S.; Parlow, J. J. Polymer-Assisted Solution-Phase (PASP) Suzuki Couplings Employing an Anthracene-Tagged Palladium Catalyst. *J. Org. Chem.* **2003**, *68* (25), 9678–9686.

[5] Glink, P. T.; Oliva, A. I.; Stoddart, J. F.; White, A. J. P.; Williams, D. J. Template-Directed Synthesis of a [2]Rotaxane by the Clipping under Thermodynamic Control of a Crown Ether Like Macrocycle Around a Dialkylammonium Ion. *Angew. Chem. Int. Ed.* **2001**, *40* (10), 1870–1875.

[6] Cantrill, S. J.; Fyfe, M. C. T.; Heiss, A. M; Stoddart, J. F.; White, A. J. P; Williams, D. J. Tribenzo[27]crown-9: A New Ring for Dibenzylammonium Rods. *Org. Lett.* **2000**, *2* (1), 61–64.

[7] Sheldrick, G. M. *SADABS: Program for Empirical Absorption Correction of Area Detector Data*, University of Göttingen, Göttingen, Germany, 1996.

[8] Sheldrick, G. M. *SHELXTL 5.10 for Windows Structure Determination Software Programs*, Bruker Analytical X-ray Systems, Inc., Madison, Wisconsin, USA, 1997.
